# Interfacial Engineering of Soft Matter Substrates by Solid-State Polymer Adsorption

**DOI:** 10.1021/acsami.4c06182

**Published:** 2024-06-12

**Authors:** Wenyang Xu, Oliver Werzer, Panagiotis Spiliopoulos, Karl Mihhels, Qixiang Jiang, Zhuojun Meng, Han Tao, Roland Resel, Tekla Tammelin, Torbjörn Pettersson, Eero Kontturi

**Affiliations:** † Department of Bioproducts and Biosystems, School of Chemical Engineering, Aalto University, P.O. Box 16300, FI-00076 Aalto, Finland; ‡ Department of Fibre and Polymer Technology, KTH Royal Institute of Technology, Teknikringen 56, SE-10044 Stockholm, Sweden; § Laboratory of Natural Materials Technology, Åbo Akademi University, FI-20500 Turku, Finland; ∥ Joanneum Research, Institute for Sensors, Photonics and Manufacturing Technologies, Franz-Pichler-Strasse 30, 8160 Weiz, Austria; ⊥ Polymer and Composite Engineering (PaCE) Group, Institute of Materials Chemistry, Faculty of Chemistry, University of Vienna, Währinger Straße 42, A-1090 Vienna, Austria; # Institute of Solid State Physics, NAWI Graz, Graz University of Technology, Petersgasse 16, 8010 Graz, Austria; ¶ Biomass Processing and Products, VTT Technical Research Centre of Finland Ltd., FI-02044 Espoo, Finland; ∇ Wallenberg Wood Science Centre, KTH Royal Institute of Technology, Teknikringen 56, SE-10044 Stockholm, Sweden

**Keywords:** solid-state adsorption, Guiselin layer, soft matter, cellulose, nanolayer adsorption, surface chemistry

## Abstract

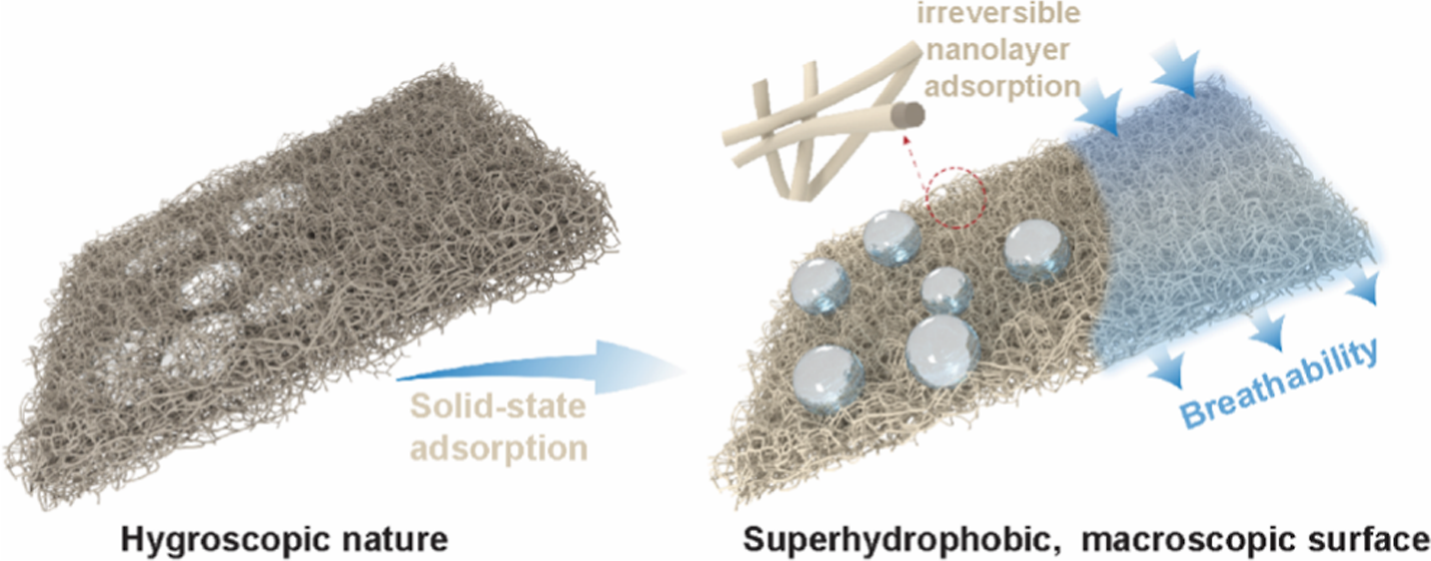

Polymer coating to substrates alters surface chemistry and imparts bulk material functionalities with a minute thickness, even in nanoscale. Specific surface modification of a substate usually requires an active substrate that, e.g., undergoes a chemical reaction with the modifying species. Here, we present a generic method for surface modification, namely, solid-state adsorption, occurring purely by entropic strive. Formed by heating above the melting point or glass transition and subsequent rinsing of the excess polymer, the emerging ultrathin (<10 nm) layers are known in fundamental polymer physics but have never been utilized as building blocks for materials and they have never been explored on soft matter substrates. We show with model surfaces as well as bulk substrates, how solid-state adsorption of common polymers, such as polystyrene and poly(lactic acid), can be applied on soft, cellulose-based substrates. Our study showcases the versatility of solid-state adsorption across various polymer/substrate systems. Specifically, we achieve proof-of-concept hydrophobization on flexible cellulosic substrates, maintaining irreversible and miniscule adsorption yet with nearly 100% coverage without compromising the bulk material properties. The method can be considered generic for all polymers whose *T*_g_ and *T*_m_ are below those of the to-be-coated adsorbed layer, and whose integrity can withstand the solvent leaching conditions. Its full potential has broad implications for diverse materials systems where surface coatings play an important role, such as packaging, foldable electronics, or membrane technology.

## Introduction

Modifications to solid surfaces of any material are performed to alter their interactive properties: the response to, e.g., solvent or light exposure, or to make it more compatible in a given matrix. Traditional tools in surface modification utilize chemical reactions in either solvent or gas/vapor/plasma medium.^1−5^ In most cases, the surface modifications must be tailored specifically for each material according to their chemistry and morphology. Soft materials pose an added challenge because of their susceptibility to harsh modification conditions, e.g., at high temperatures.

This study aims at introducing a generic approach of surface modification of soft materials with irreversible polymer adsorption in the solid state. Solid-state adsorption of polymers is based on a well-investigated phenomenon: a strongly adsorbed, so-called Guiselin layer of just 0.5–10 nm thickness emerges on a substrate surface upon annealing of a sufficiently mobile polymer above its glass transition temperature (*T*_g_) or melting point (*T*_m_) and subsequent leaching of the excess (nonadsorbed) polymer with a good solvent.^6−9^ Solid-state adsorption is pronouncedly distinct from a weakly attached and poorly covering adsorbed layer from a solution, i.e., the more familiar form of polymer adsorption.^10,11^ Even though there are numerous open questions about the fundamentals of Guiselin layer formation, e.g., in context of thermodynamics and nanoconfinement relating to polymer properties,^12−15^ the generic deposition method is effortless and has several advantages over the existing techniques for surface modification.^16^ For example, it does not need a special reactor or a reactive surface like chemical vapor deposition (CVD), it does not require specific expertise like deployment of specific organic reactions, it is not restricted to layers of alternating binding like layer-by-layer deposition, and it is more selective than plasma treatments. Because of the fundamental mindset in the relevant research,^8,9,14^ Guiselin layers have hitherto been exclusively deposited on hard inorganic substrates, such as silicon wafers, without paying much attention to their specific influence on materials construction.^16^ Here, we introduce Guiselin layers to the realm of soft materials science, specifically demonstrated as a modification tool for wood-based cellulosic surfaces, although this concept is not confined to a specific set of substrates.

We stress that molten or rubbery polymers—although often termed liquid polymers—are generally not considered to be in liquid state per se, as they retain many conventional solid-state properties despite a change in their viscoelastic response.^17^ Therefore, we have opted for the term solid-state adsorption when referring to the Guiselin layer deposition.

We have chosen cellulose materials as the model substrates here because of their relevance to modern materials research. Particularly nanocellulose has been highlighted as a game changer in sustainable materials science,^18−22^ but many fundamental challenges are impeding the development of genuine applications.^23−25^ Water/vapor susceptibility of cellulose^26^ and inherent incompatibility of nanocellulose in composites^27,28^ are examples of such impediments. Here, surface modification is among the key challenges: from compatibility issues in composites and selectivity in membranes to responsive features in smart materials—the interfacial interactions play a role everywhere. The state-of-the-art of modification involves applying the tools of organic chemistry to introduce specific functional groups on the nanocellulose surface.^28−32^ The relevant methods often suffer from accessibility issues, harsh reaction conditions, and poor control over the substitution. Furthermore, these methods are often not scalable and may be economically unrealistic. Another traditional approach is to coat a thick plastic film on the cellulose surface—such as the ∼100 μm thick polyethylene film in liquid packaging cartons to prevent water infiltration—but this is not viewed as a benign technology in a modern sustainable society.

In this report, we first show how Guiselin layers of polystyrene (PS) can be deposited on flat, planar model cellulose films. PS was chosen as a polymer for the fundamental study, as its behavior in Guiselin layer deposition has been investigated more than that of any other polymers. In the follow-up section, we demonstrate Guiselin layer deposition of polylactic acid (PLA) on cellulosic nonwoven, cellulosic fiber paper network, and free-standing networks of cellulose nanofibers (CNFs) termed cellulose nanopapers or nanocellulose films.^33,34^ With their intrinsic properties, such as high mechanical strength and transparency, nanopapers have been proposed for multiple materials applications, including smart packaging, membranes, and organic electronics. As with any cellulosic material, however, their strength is impaired in the presence of water, preventing their realistic perusal in many of these applications. Solid-state adsorption, as demonstrated in this study, presents the potential to replace, for example, the plastic films in liquid packaging with a nanosized layer of a biobased and compostable alternative (PLA), reducing the amount of required material by 5 orders of magnitude. In principle, the method of Guiselin layer deposition is applicable to any polymer/substrate combination where *T*_g_ or *T*_m_ of the coated polymer is below the thermal degradation temperature of the substrate and where the substrate can withstand the solvent leaching conditions.

## Results and Discussion

**Figure 1:**
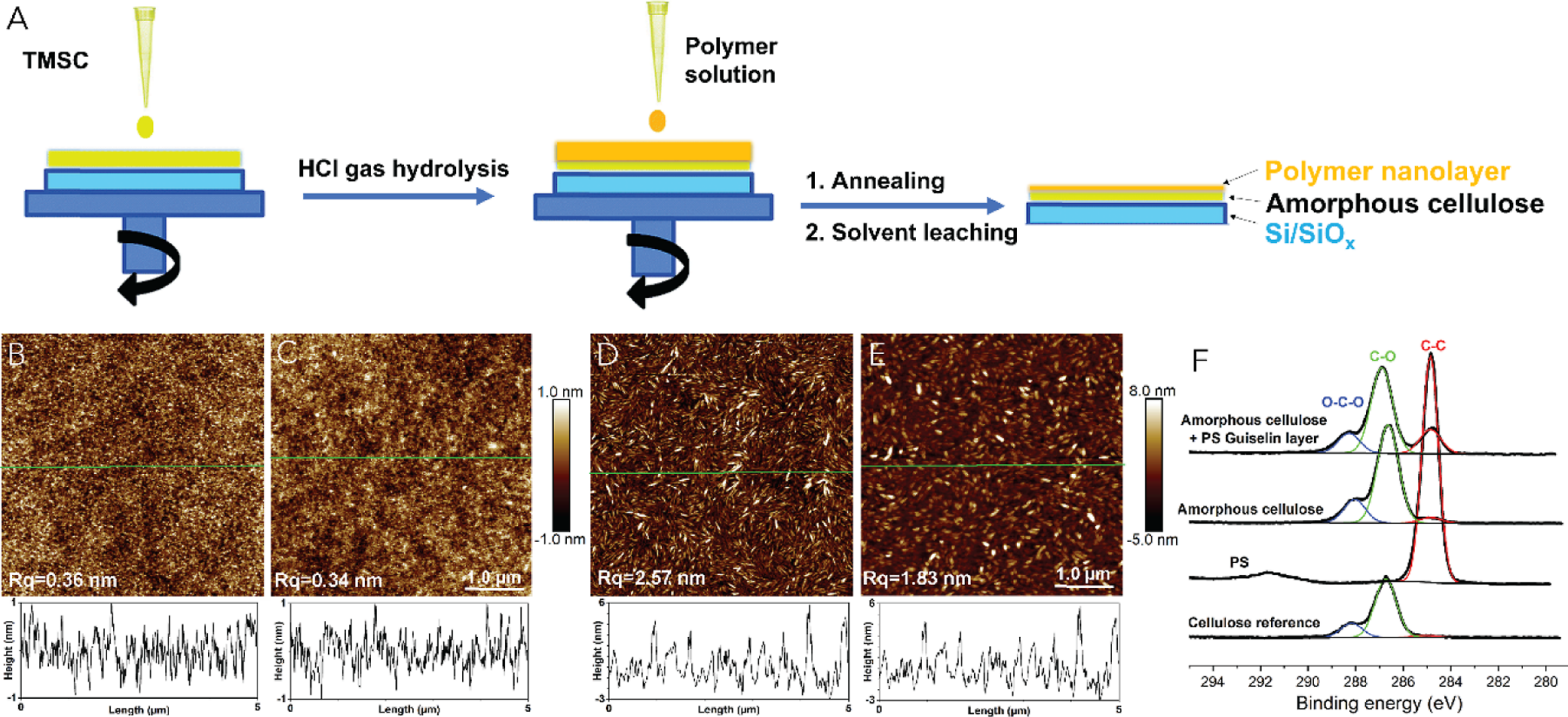
(A) Schematic illustration of solid-state adsorption process from creating an ultrathin amorphous cellulose film using spin-coating and regeneration via HCl gas hydrolysis of TMSC [synthesis of trimethylsilyl cellulose (TMSC) referring to Supporting Information], to depositing polymer layer (thickness larger than few radii of gyration), followed by annealing above glass transition temperature before rigorous solvent leaching. Surface morphological and chemical changes of cellulose substrates before and after solid-state adsorption of PS. (B–E) AFM height images (5 × 5 μm^2^) with height profiles (from green line) of representative films of regenerated amorphous cellulose film, regenerated amorphous cellulose after the solid-state adsorption process of PS, spin-coated CNC film, and solid-state adsorption of PS with underlying CNCs, respectively. (F) High-resolution C 1s XPS data of reference samples (pure cellulose and polystyrene), amorphous cellulose as well as amorphous cellulose after polymer solid-state adsorption (amorphous cellulose + PS Guiselin layer), where black curves are raw experimental spectra with the baseline and other colors are from spectra deconvolution.

The preparation of solid-state adsorption of polymers on a cellulosic surface is illustrated in Figure 1A. Because soft materials have never really been used as substrates for solid-state adsorption earlier, studies were first carried out with smooth ultrathin model films of cellulose, namely, regenerated amorphous cellulose and spin-coated cellulose nanocrystals (CNCs) which were subjected to Guiselin layer deposition with PS. AFM images in Figure 1 show how the topographies of cellulose substrates before and after solid-state adsorption of monodispersed PS remain similar to each other. Amorphous cellulose represents a smooth featureless film with a root-mean-squared roughness (*R*_q_) of 0.36 nm (Figure 1B) with minimal effect from the adsorbed Guiselin layer of PS where the *R*_q_ is 0.34 (Figure 1C). By contrast, CNCs are conspicuous rod-like nanocrystals that form an anisotropic network upon spin coating (*R*_q_ = 2.57 nm, Figure 1D). Intriguingly, a slight visual change was observed in the film morphology upon Guiselin layer deposition, exhibiting a smaller *R*_q_ of 1.83 nm (Figure 1E). According to solid-state adsorption scenario on hard matter, the morphological changes caused by an adsorbed Guiselin layer on a smooth substrate are minimal, unless dewetting (i.e., film rupture) occurs.^9,35−40^ As such, the results in Figure 1 are in line with the previously published data.

XPS analyses expose hard evidence on the presence of PS on cellulose, as shown in the high-resolution C 1s emission of Figure 1F. All carbons in cellulose are bound to at least one oxygen atom (Supporting Information, Figure S1), implying that the small proportion of the oxygen-less (C–C) contribution within pure cellulose is generally from impurities.^41^ With the deposited Guiselin layer (amorphous cellulose + PS Guiselin layer, Figure 1F), the C–C contribution is vastly increased. As suggested from adsorption on the surface of hard matter, the occurrence of the adsorption is supposed to be mainly driven by the monomer pinning mechanism along the decreasing adsorption sites.^42,43^ Moreover, as a microporous substrate, amorphous cellulose is following the changes of fiber aggregation or fibrillation with enlarging the micro- and mesopores between regenerated fibrils along the annealing process,^44^ which may promote the chain monomer pinning process.

**Figure 2:**
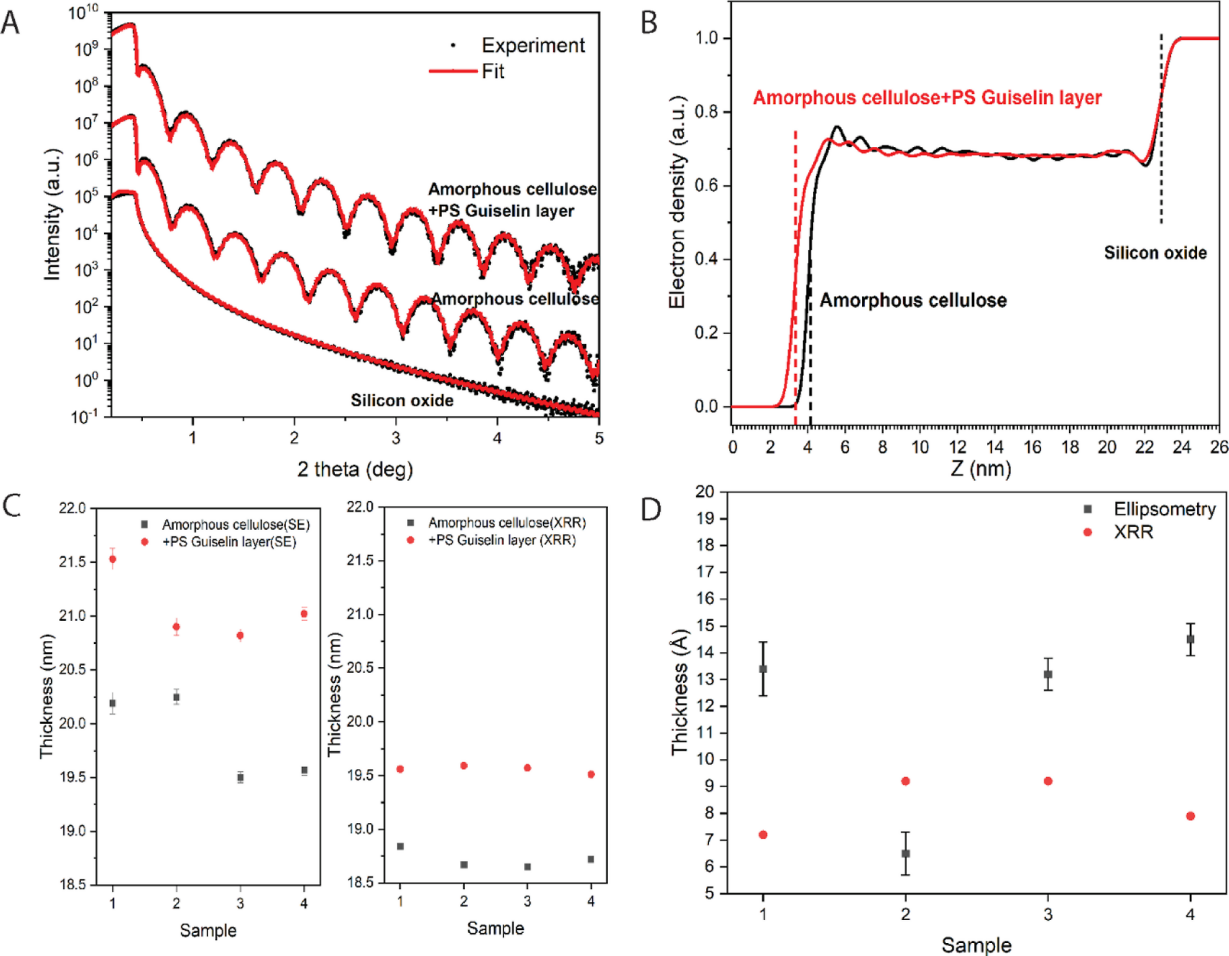
Thickness evaluation of the adsorbed PS layer with amorphous cellulose substrate. (A) X-ray reflectivity curves of silicon oxide substrate and amorphous cellulose film before and after solid-state adsorption. Black dots denote the experimental data; red full lines are the model independent fitting results. (B) Electron density profiles determine the thickness change of cellulose film before and after solid-state adsorption. The surface roughness at the interfaces can be referred as the *y*-axis value of the electron drop midpoint. (C) Thickness evaluation of the cellulose film by both SE and XRR, where the exact same four replicates were measured before and after solid-state adsorption. The error bar for ellipsometry was obtained from standard deviation of different 9-point measurements. The accuracy for the XRR thickness measurements is ±1%. (D) Thickness values of the adsorbed PS layer as calculated from ellipsometry as well as X-ray reflectivity data of the same four samples from (C).

The thickness change of the adsorbed PS layer on the cellulose substrate is pivotal for materials applications and was shown to be in the range of 1 nm as determined by XRR and ellipsometry (Figure 2 and Table 1). Intriguingly, the thickness of the adsorbed polymer layer from the same process exhibited merely 20% of the reported thickness of a PS Guiselin layer on hard matter, i.e., ca. 5.5 nm on silica.^40^ Even higher layer thickness values of up to 10 nm have been reported on silica with PS grades of different *M*_w_ distributions.^9,45^Figure 2A shows the XRR data of the representative amorphous cellulose before and after solid-state adsorption of PS on thick silica (around 150 nm), revealing a typical reflectivity trajectory of a homogeneous thin film at a solid planar surface. The adsorbed PS adsorption yields a layer thickness of ca. 0.9 nm as computed from the electron density profile (Figure 2B), which is well in line with the thickness range from ellipsometry measurements for the same samples (Figure 2C,D) and for other replicate samples (Supporting Information, Figure S2). It is also noteworthy that a good quality fit was obtained between the experiments and the model from ellipsometry (Figure S3). The deposited amorphous cellulose layer is smooth (*R*_q_ = 0.45 nm after regeneration, Supporting Information, Figure S4) with a thickness at ca. 20 nm, which is fully reproducible and in agreement with the previous study of amorphous cellulose deposition using the same process.^44^

**Table 1:** Roughness and Water Contact Angles [Static, Advancing (θ_a_), and Receding (θ_r_) Contact Angles] of the Samples: Model Amorphous Cellulose Surface before and after Polymer Adsorption Using PS and PLA, as Well as Cellulose Nanopaper before and after PLA Guiselin Layer Formation

samples	roughness (*R*_q_)^a^/nm	thickness/nm	thickness of Guiselin layer^b^/nm	static contact angle/deg	θ_a_, θ_r_/deg
amorphous cellulose on silica	0.36	19.8 ± 0.34^SE^, 18.7 ± 0.07^XRR^	N.A.	28.0 ± 0.5	41.3 ± 1.4, 28.0 ± 2.0
amorphous cellulose + PS Guiselin layer	0.33	21.06 ± 0.27^SE^, 19.56 ± 0.03^XRR^	2.06	71.3 ± 0.5	90.2 ± 3.3, 72.2 ± 0.1
PLA spin coated on silica		49.10 ± 0.43^SE^	N.A.	73.4 ± 0.6	76.8 ± 0.8, 58.7 ± 0.3
PLA Guiselin layer on silica	0.22	6.29 ± 0.22^SE^	6.29	74.9 ± 0.4	76.1 ± 0.6, 59.4 ± 0.2
amorphous cellulose + PLA Guiselin layer	0.31	21.51 ± 0.30^SE^ (19.56 ± 0.24)	2.75	76.5 ± 0.1	77.8 ± 0.5, 60.2 ± 0.5

a Root mean square roughness (*R*_q_) is obtained from AFM height images. Thickness values in average of at least four samples were characterized by using SE and XRR as indicated with superscripts. The average thickness of amorphous cellulose before PLA Guiselin layer deposition is shown in bracket.

b Thicknesses of Guiselin layer take considerations of thickness difference before and after Guiselin layer formation from SE as well as the possible thickness change of the amorphous cellulose during treatment.

However, a minute thickness change of the amorphous layer should still be considered along each step of Guiselin layer deposition processes including the initial PS layer deposition, annealing, and solvent leaching. As is clearly depicted (Supporting Information, Figure S5), the thickness of the amorphous cellulose merely against toluene washing decreased 0.2–0.3 nm, possibly due to the rearrangement of amphiphilic cellulose molecules against a hydrophobic solvent.^46^ Moreover, the thickness of amorphous cellulose decreases by 3% of its original thickness upon annealing at 105 °C, according to a previous study.^44^ Therefore, the thickness of the PS Guiselin layer should take into consideration the thickness variation of the underlying cellulose layer during the whole process on top of the computed thickness from the difference of the organic layer above silicon oxide layer before and after solid-state adsorption. In this sense, for the amorphous cellulose film of 20 nm, a thickness value of ca. 0.8–0.9 nm is rational to top up the computed thickness value from XRR and ellipsometry, resulting in an overall estimated thickness of ca. 2 nm (Table 1).

Nevertheless, both XRR and ellipsometry here serve as ex situ methods where a real time thickness change of the amorphous cellulose layer can hardly be tracked for determining the real thickness of the adsorbed polystyrene. To unveil the genuine thickness of the adsorbed layer, a contrast matching by neutron reflectivity would be an enticing approach for studying the interfaces between cellulose and the adsorbed polymers.^47^ In addition, the surface roughness of the amorphous cellulose with and without PS adsorption can be estimated from the electron density distribution at the air–film interface and is about 0.4 nm. Furthermore, as indicated in Supporting Information, Figure S6, the confined nanocoating on cellulose is stable and firmly attached on the cellulose surface even against rigorous solvent leaching up to 9 days. Indeed, there is no difference in the thickness of the layers after rinsing for 1 h and 9 days. Thus, it is conclusive that 1 h solvent leaching is sufficient to remove the nonadsorbed polymers. Collectively, the solid-state adsorption occurs with a change of surface chemistry in proximity to a substrate surface, while the surface morphology barely changes.

Through solid-state adsorption of PS, a hydrophilic cellulose surface was transformed to a hydrophobic surface (Table 1 and Supporting Information, Figure S7). The static water contact angle of ca. 28° for amorphous cellulose represented a hydrophilic surface, which indicates a successful regeneration from hydrophobic TMSC via HCl vapor.^48^ A ∼90° static contact angle of pristine PS and PS Guiselin layer on silica (Supporting Information, Figure S7B) is what can be reached with a pure homogeneous PS surface and after PS adsorption on plain silica with full surface coverage, according to our recent reports.^40^ A stable water drop with a higher contact angle of ∼72° is observed after solid-sate adsorption comparing with a lower contact angle (θ_a_/θ_r_ = 36°/24°) from solution-based polymer adsorption after washing nonadsorbed polymers as reported by Kontturi et al.^11^ However, the cellulose substrate after PS Guiselin layer deposition shows the pinning of the liquid front (θ_a_/θ_r_ = 90°/72°) with the contact angle hysteresis at ca. 18°. This reflects a possible chemical inhomogeneity on the surface by contrast to the adsorbed PS on silica with full coverage shows a low contact angle hysteresis (ca. 7°) in our recent report.^40^ Nevertheless, according to the Cassie–Baxter equation, solid-state adsorption at ca. 64% surface coverage of PS over the amorphous cellulose surface is significantly higher than the direct adsorption from a PS solution, achieving less than 10% surface coverage.^11^ Therefore, the PS adsorption via solid-state adsorption renders a steady approach to achieve surface modification with higher stability and higher surface coverage of films with thickness in the nanometer range compared to adsorption via polymer solution.

### Solid-State Adsorption of Polylactic Acid to Modify Macroscopic Cellulosic Substrates

**Figure 3:**
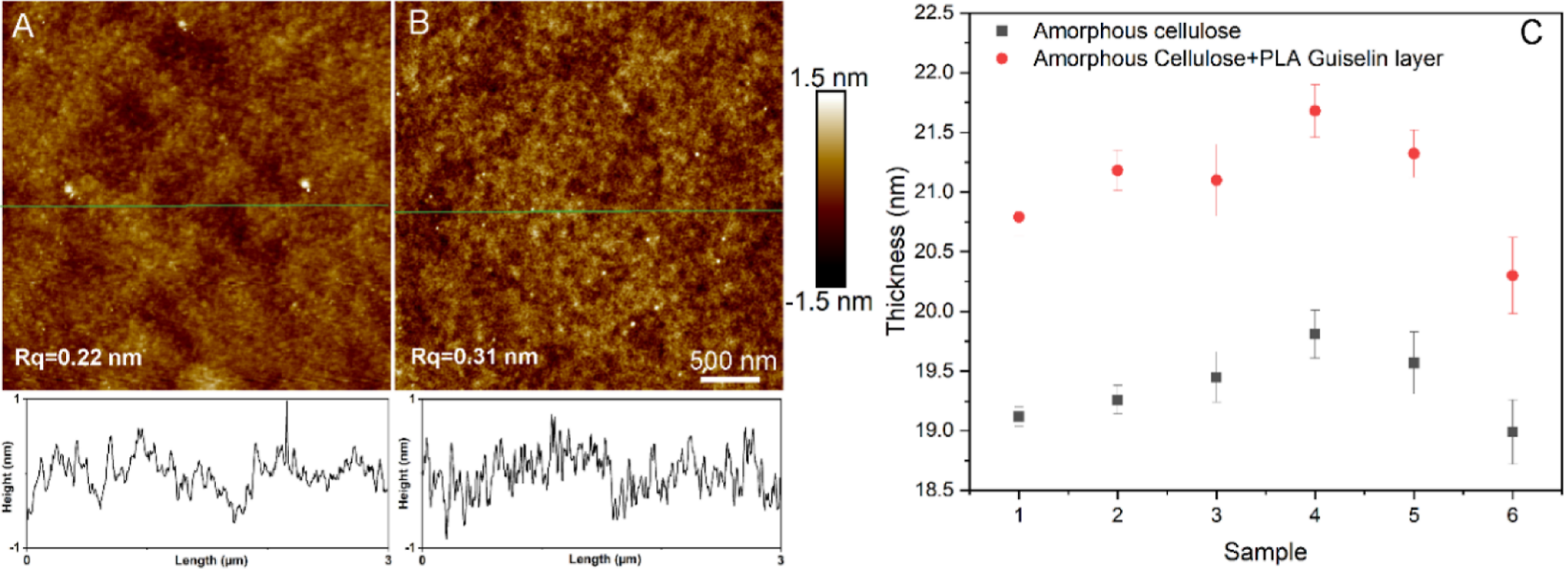
Surface morphological change and thickness evaluation of cellulose substrates before and after the solid-state adsorption of PLA. (A,B) AFM height images and height profiles (from green lines) of representative samples of PLA Guiselin layer on silica substrates and on the amorphous cellulose model surface. (C) Thickness evaluation of PLA Guiselin layer on amorphous cellulose replicates via spectroscopic ellipsometer concluding an average thickness difference comparing before and after PLA Guiselin layer of ca. 1.7 nm. Here, thickness of six replicates was measured and compared before and after solid-state adsorption of PLA. The error bar was obtained from standard deviation of different 9-point measurements.

Due to the nature of bioresource and biodegradability, the PLA Guiselin layer would serve as a sustainable approach to modifying cellulose substrates. Similar to the PS Guiselin layer, no morphological changes can be discerned from the AFM images of PLA Guiselin layer on the plain silica surface and amorphous cellulose surface (Figure 3A,B). In turn, the adsorbed PLA Guiselin layer exhibited similar water repellency of the modified silica surface and amorphous cellulose surface when compared to pristine PLA after spin coating (Table 1). This suggests a successful PLA Guiselin layer formation on the silica and amorphous cellulose surface. Most importantly, the PLA Guiselin layer appears to possess a full surface coverage on both silica and amorphous cellulose according to similar surface heterogeneity as revealed from a quasi-static water contact angle. Intriguingly, the PLA Guiselin layer also presented a slightly thicker layer, i.e., 6.29 nm in average on the plain silica surface (SE fitting of representative sample in Supporting Information, Figure S8) and 2.75 nm on the amorphous cellulose surface (Figure 3C and Supporting Information, Figure S9), in comparison with the PS Guiselin layer on both planar silica (5.50 nm) and amorphous cellulose surface (2.06 nm) from the ellipsometry study with taking consideration of thickness change of amorphous cellulose layer (Figure 3, and Table 1). The irreversibly adsorbed PLA Guiselin layer is around three times thicker than the PLA (*M*_n_, 12.9 kDa) monolayer thickness of 0.62 nm as revealed by Langmuir–Blodgett (LB) deposition.^49^ (We stress that even an LB-deposited polymer layer is not a genuine monolayer as it has a degree of overlap but it is, to our knowledge, the thinnest uniform layer that one can achieve from a polymer.) Essentially, adsorption requires mass transport toward the interface and the optimization of chain conformations to achieve the highest gain in free energy on densification.^50^ Unlike the typical physical adsorption of a macromolecule onto an interface in the liquid or gas phase in an exothermic process, solid-state adsorption entails a molecule chain pinning to an empty site at the interface through chain mobility facilitated by annealing. It was reported that low annealing temperature causes a less efficient transport of chains toward the interface.^9^ Therefore, the difference of thickness between PS and PLA upon adsorption (i.e., slightly thicker for PLA compared to PS) might be associated with the applied annealing temperature difference against the *T*_g_ of the applied polymers. The annealing temperature of 120 °C for PLA is around 70 °C higher than that of its *T*_g_, whereas the annealing temperature of PS is only 50 °C above that of its *T*_g_. The enhanced Guiselin layer thickness may also associate with the differences of molecular weight and polarity of the polymers, where more evidence would be required for solid-state adsorption process to draw such conclusions. It was also identified that high-temperature flow against polymer *T*_g_ can efficiently drive polymer melts and glasses toward a more stable state,^12^ which may be correlated to the higher surface coverage of PLA Guiselin layer in comparison to the PS Guiselin layer on the amorphous cellulose film. Therefore, a quantitative understanding of the thickness scale of the perturbation to a soft material surface affecting interfacial properties would improve the design of nanostructured and functional polymer materials exploiting the enhanced properties of polymers at the nanoscale.

**Figure 4:**
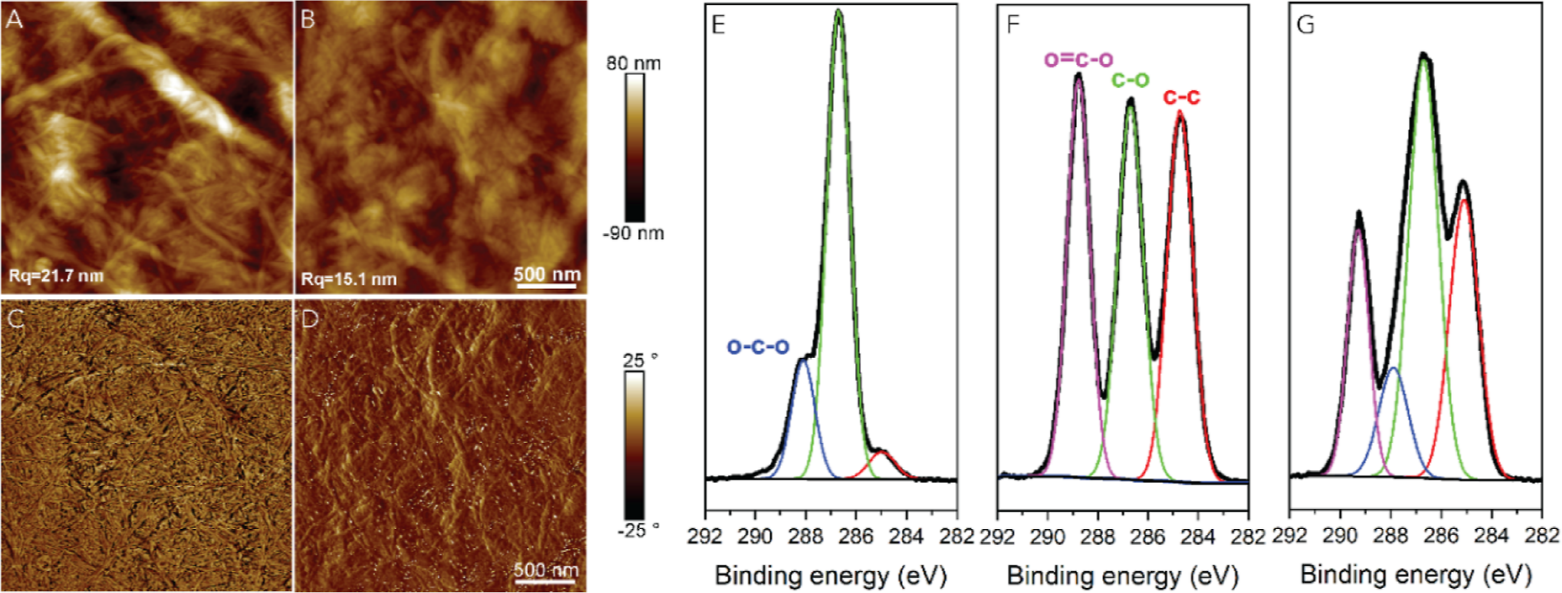
Changes in the surface morphology and surface chemistry of cellulose nanopapers before and after solid-state adsorption of PLA. (A,B) AFM topography images of pristine cellulose nanopaper and cellulose nanopaper with PLA Guiselin layers. (C,D) AFM phase images of pristine cellulose nanopaper and cellulose nanopaper with PLA Guiselin layers. (E–G) High-resolution C 1s XPS spectra of samples of cellulose nanopaper, cellulose nanopaper after PLA drop casting from chloroform solvent, and cellulose nanopaper with the PLA Guiselin layer, where black curves are raw experimental spectra with the baseline and other colors are from spectra deconvolution.

**Figure 5:**
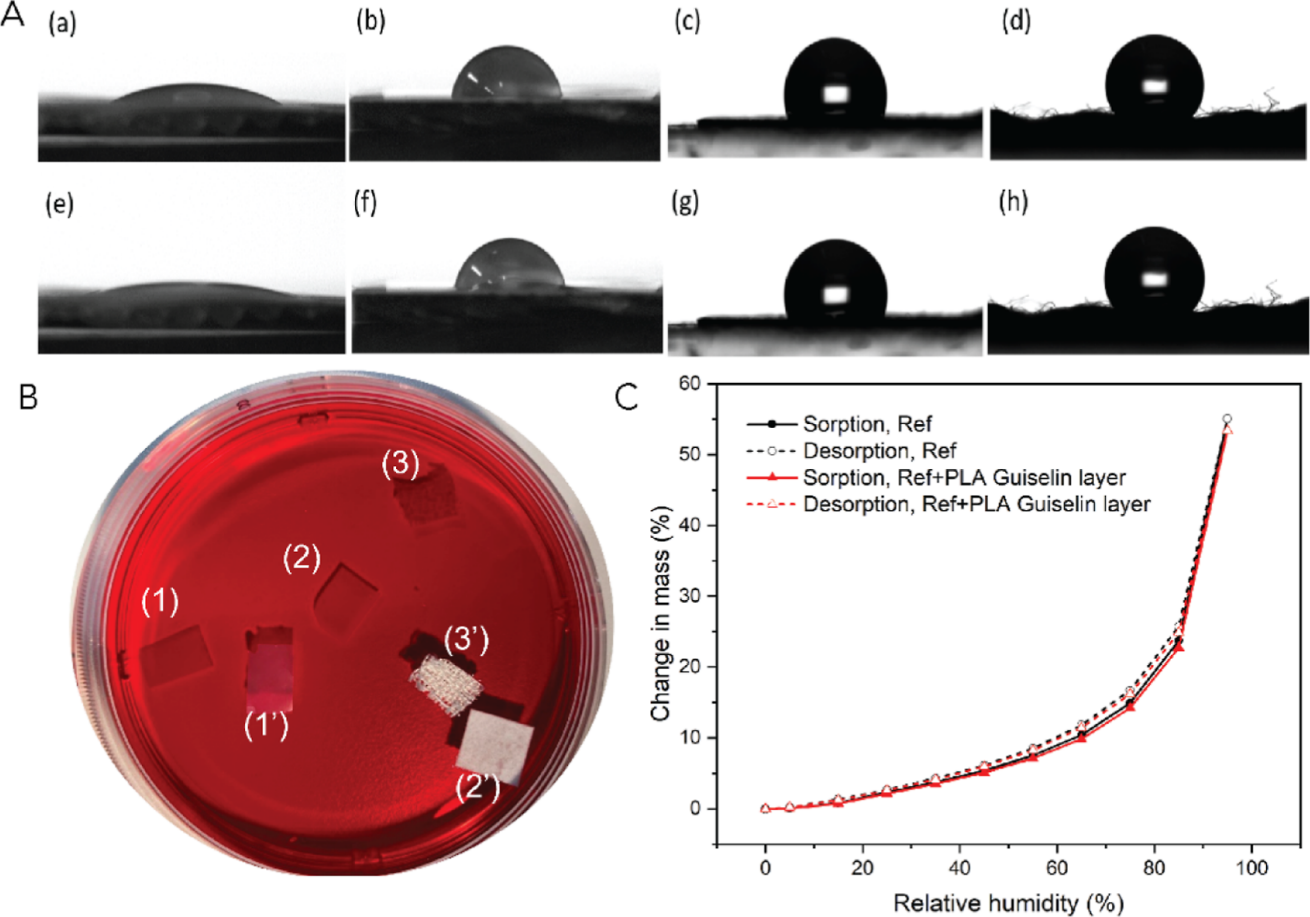
Macroscopic cellulose substrates bulk water repellency and water vapor breathability after solid-state adsorption of PLA. (A) Static contact angle after placing water drop on the substrate surface at *t* = 0 s of (a) pristine cellulose nanopaper, (b) nanopaper with the PLA Guiselin layer, (c) cellulose paper with the PLA Guiselin layer, as well as (d) cellulose fabrics with the PLA Guiselin layer. Static contact angle after placing a drop on the substrate surface after *t* = 60 s of (e) pristine cellulose nanopaper, (f) nanopaper with the PLA Guiselin layer, (g) cellulose paper with the PLA Guiselin layer, as well as (h) cellulose fabrics with the PLA Guiselin layer. (B) Absorbing dyed water of the pristine samples of (1) pristine cellulose nanopaper, (2) paper, and (3) fabrics; floating on top of water surface of the samples of (1′) nanopaper + PLA Guiselin layer, (2′) cellulose paper + PLA Guiselin layer, as well as (3′) fabrics + PLA Guiselin layer. The floating status has been steady for 3 weeks. (C) Dynamic vapor sorption of nanopaper without treatment and after deposition of PLA Guiselin layer.

Due to its full coverage on the cellulose model surface, the PLA Guiselin layer was further attempted to adopt onto a macroscopic cellulose film, i.e., cellulose nanopaper. Prior to annealing, a thick PLA layer was deposited to a nanopaper surface by drop casting, as indicated by the lack of cellulose OH groups in an ATR-FTIR spectrum (detection limit of ca. 1 μm, Supporting Information, Figure S10). After PLA Guiselin layer formation, the nanopaper presents a similar isotropic network of individual nanofibers (Figure 4B) compared to that of a pristine nanopaper (Figure 4A), according to AFM topography. However, the nanopaper with PLA Guiselin layer exhibited less surface heterogeneity according to the phase image (Figure 4D) compared to that of pristine nanopaper where interfibrillary spaces were clearly visible (Figure 4C). It is also noteworthy that the surface roughness has decreased from 21 to 15 nm after PLA Guiselin layer adsorption. Concerning the XPS spectra, after rigorous solvent leaching of the nonadsorbed polymers, the cellulose nanopaper surface (Figure 4G) showed a conspicuous chemical feature of PLA (Figure 4F) with a discernible band increase of O=C–O and C–C compared with the pristine cellulose nanopaper (Figure 4E). The FTIR spectra of cellulose nanopaper before and after PLA adsorption (Supporting Information, Figure S10) supports the surface chemical change with a band appearing at 1750 cm^–1^, corresponding to C=O stretching of PLA. Macroscopically, a nonhygroscopic cellulose nanopaper was achieved after solid-state adsorption of PLA (Figure 5). The water contact angle of cellulose nanopaper with the PLA Guiselin layer was maintained at around 81° for at least 60 s benefiting from a high surface coverage of the PLA Guiselin layer and its rough surface (Supporting Information, Table S1, and Figure 5 panel A), which indicates a homogeneous thin surface layer with a high surface coverage of the adsorbed layer. This is well in line with the earlier observations on cellulose model films that the Guiselin layers of PLA renders a full surface coverage on the amorphous cellulose surface.

### Solid-State Polymer Adsorption as a Modular Tool for Surface Modification

Aside from the studies on homogeneous cellulose nanopaper, the PLA Guiselin layer was further attempted to be manifested on heterogeneous, fibrous, and macroscopic cellulosic substrates including filter paper and fabrics. The good water repellence of bulky cellulose paper and cellulose-based fabrics (Figure 5, panel A) demonstrates the wide applicability of solid-state polymer adsorption even with the morphological complexity of soft materials. As also shown in Supporting Information, Videos S1, S2 and S3, water penetration to both cellulose paper and fabrics was highly prohibited, benefiting from the surface polymer adsorption and bulk roughness. It even renders a close to superhydrophobic properties of cellulose-based fabrics (contact angle of ca. 150° of fabrics + PLA Guiselin layer, Figure 5 panel A), partially due to the roughness effects of the surface. Nevertheless, compared to their hygroscopic nature, all the cellulose-based substrates maintain their pristine state without notably absorbing water in up to 3 week periods [Figure 5 panel B, as compared (2′) and (3′) with (2) and (3)]. Most importantly, the vapor transmission of the PLA Guiselin layer-modified nanopaper was comparable to the unmodified nanopaper as supported by the water vapor transmission tests (Supporting Information, Table S2). The bulk water vapor uptake in the nanopaper with the PLA Guiselin layer also stays virtually unaffected compared to nanopapers without modification as determined by dynamic vapor sorption (Figure 5 panel C). Therefore, solid-state adsorption offers an enticing approach to fabricate breathable substrates in addition to good bulk water repellence. Moreover, cellulose nanopapers after surface modification with a PLA Guiselin layer maintain their intrinsic transparency (Supporting Information, Figure S11). Solid-state adsorption would improve application efficacy in where the application environments are often humid, moist, or wet, and enhance interfacial compatibilization for further deposition of hydrophobic conductive polymers. Therefore, solid-state adsorption to cellulose nanopaper would supply a promising group of platform materials in, but not limited to a variety of applications, e.g., organic photovoltaic and optical devices, etc.^51,52^ All in all, solid-state adsorption to the cellulose surface paved a magnificent approach for generically constructing functional surfaces.

Collectively, solid-state adsorption offers an alternative realistic strategy for surface functionalization in view of generic applications, with respect to other surface modification strategies. On one hand, chemical modification of nanocellulose surfaces via polymer grafting is often applied to individual fibers for implementing efficacies, for instance, achieving better compatibility with composite matrices.^53^ As an alternative approach to intricate chemical reactions, physical adsorption highlights binding functional substances also on the individual fiber surface as a result of van der Waals forces,^46,54,55^ electrostatic forces,^56−59^ and hydrogen bonding,^60−62^ in demanding need of solvent presence. In the above-mentioned two process categories, the virgin robust hydrogen bonding of cellulose fibers is sacrificed, which precludes the strength of the interfibrillar network regarding material development, to a certain extent. Even worse, the breathability of the interfibrillar network, i.e., vapor accessible areas of the fibrous, usually suffers a loss after direct individual fiber chemistry manipulation.^63^ A good balance of liquid water repellence and water vapor sorption/permeation is usually obtained through a heavy manipulation process.^64^ On the other hand, as an age-old affair, for instance, the use of sizing chemicals or extrusion-based plastic films is an industrially facile process to alter surface properties of cellulosic materials. However, those methods offering tens of micrometer coating layers overwhelms the beneficial of certain intrinsic properties and even causes other detriments such as microplastic formation.^65^ A magnificent attempt of polymer adsorption from aprotic solvents to macroscopic cellulose surface, i.e., cellulose nanopaper, establishing a generic way of surface modification in a bulk manner, yet failed with high adsorption efficacy.^11^ Therefore, standing on an exclusive nanolayer and full surface coverage adsorption, solid-state adsorption is an asset of modifying surfaces of soft matter, i.e., cellulosic substrates, without interfering to the interior.

## Conclusions

We have presented a generic approach, i.e. solid-state polymer adsorption, to modify a soft material surface. Proof-of-concept was provided by using PS and PLA as the model polymers and various cellulose-based materials as model substrates. By bringing a thick polymer layer in contact with an interface, followed by annealing and solvent leaching, an ultrathin layer was irreversibly adsorbed on the cellulose surface, completely altering its surface chemistry. Planar model surfaces of cellulose were utilized, together with ellipsometry and XRR, to reveal minute thickness values of 2 nm for PS and 2.7 nm for PLA after solid-state adsorption, while contact angle data exposed virtually a full coverage for the ultrathin layers. A number of bulk cellulosic substrates were further employed to demonstrate the robustness of our approach. Textile fabric, filter paper, and cellulose nanopaper were all made water repellent at the surface upon solid-state adsorption of PLA, while retaining their bulk properties. For example, cellulose nanopaper exhibited a hydrophobic surface with a water contact angle of >80°, while preserving their transparency and vapor-breathing nature. Such properties will be beneficial in, for example, applications concerning textiles or insulation. Moreover, the approach is a generic one: it is applicable to all polymer/substrate systems where the glass transition or melting point of the polymer is below the degradation temperature of the substrate and where the substrate is able to withstand the solvent rinsing conditions of the excess polymer. We believe that precisely the generic nature of the approach is likely to broaden the applicability of solid-state adsorption as a surface modification method for soft materials, for example, in the fields as diverse as packaging, foldable electronics, or membrane technology.

## Materials and Methods

### Materials

Polystyrene (PS, *M*_w_ ∼524,000, *M*_n_ ∼502,000, *D̵* ∼1.04) was purchased from Sigma-Aldrich. Ingeo Biopolymer PLA 6060D (glass transition temperature, *T*_g_, 55–60 °C; *M*_n_ ∼71,700 g/mol, *D̵* ∼1.68) was purchased from NatureWorks. Toluene (99.9%) and chloroform (99.9%) were purchased from VWR Chemicals and used as received. Cellulose nanocrystals (CNCs) were prepared from ashless Whatman filter paper 1 (Whatman, GmbH, Dassel, Germany). Sulfuric acid (H_2_SO_4_), and 0.1 M sodium hydroxide (NaOH) were purchased from Sigma-Aldrich. Ethanol (Aa grade 99.5%, w/v) was purchased from the Altia Corporation (Finland). A dialysis membrane (cut off 12–14 kDa) was purchased from Spectrum Laboratories, Inc., USA. Trimethylsilyl cellulose (TMSC, *M*_w_ ∼145,460, *M*_n_ ∼71,180, *D̵* ∼2.04) was synthesized from the cellulose powder (from spruce, length of fibers: 0.02–0–15 mm, Fluka) as described below. Anhydrous LiCl (≥98.0%), dimethylacetamide (≥99.9), hexamethyldisilazane (≥99.9, HMDS), and tetrahydrofuran (≥99.5%) were purchased from Sigma-Aldrich. Methanol (100%) was purchased from VWR Chemicals and used as received. Aqueous HCl (37%) was also purchased from VWR Chemicals and was diluted to 3 M for use. Silicon wafers with a around 150 nm SiO_*x*_ layer were purchased from SIEGERT WAFER GmbH (Germany). Cellulose nanopapers from mechanically produced cellulose nanofibrils (CNFs, containing 13% glucose, 23% xylose and 0.15% methyl glucuronic acid) were kindly provided by VTT, Finland. Filter papers used for adsorption purpose were the same one as for CNC preparation. Cotton-based textile fabrics were purchased from Eurokangas Oy, Finland.

## Methods

### Preparation of Cellulosic Substrates

Cellulose nanocrystals (CNCs) were prepared from a commonly used protocol with 64 wt % sulfuric acid hydrolysis. To remove surface impurities, the CNC dispersion was freeze-dried, followed by Soxhlet extraction with ethanol for 48 h. Cellulose nanopaper was produced by using cellulose nanofibrils (CNFs). Trimethylsilyl cellulose (TMSC), the amorphous cellulose precursor, was synthesized and characterized according to a method established by Kontturi et al.^48^ More detailed preparation protocols of each cellulosic substrates were described in the Supporting Information.

### Deposition of Model Films of Cellulose

Cellulose thin film samples were prepared using a WS-650SX-6NPP/LITE spin coater (Laurell Technologies Corporation, North Wales, PA, USA). Prior to spin coating, the silicon wafers were cleansed with a three-step method. First, the wafers were sonicated with an ultrasonic bath using different solvents in an order of: Milli-Q water, isopropanol, acetone, Milli-Q water. Second, the dried wafers were cleansed in a UV ozone chamber (Bioforce Nanosciences Inc., California, USA) for a minimum of 15 min. Third, the wafers were purged with N_2_ gas to remove dusts. A volume of 10 g/L TMSC solution in toluene was dropped onto the wafer surface, after which spin coating was conducted at 4000 rpm with an acceleration of 4000 rpm/s for 90 s. The cellulose ultrathin film (thickness of 20 ± 0.5 nm) was achieved by hydrolyzing TMSC thin films using acid vapor in a vacuum-sealed desiccator with 3 M HCl for 2 min, as shown in Figure S1.^48^

For CNC thin-film preparation, the dried CNCs were immersed overnight with Milli-Q water at a concentration of 10 g/L and then tip-sonicated by Sonifier (Branson, China) for 0.5 h at 10% amplitude to achieve CNC dispersion over ice bath. The CNC thin films were obtained by spin-coating the redispersed CNCs followed by annealing at 80 °C for 10 min to enhance the film stability.^66^

### Irreversible Polymer Adsorption (Guiselin Layer Formation)

Solid-state adsorption was conducted on the cellulose substrate, i.e., ultrathin regenerated amorphous cellulose (Figure 1A) and CNC thin films by spin-coating a polymer layer (PS 20 g/L in toluene for approximately 110 nm thickness, or PLA 10 g/L in chloroform approximately 50 nm thickness), according to our previous study.^40^ The bilayer films (cellulose thin films with polymer films on top) were placed in a vacuum oven annealing for 24 h at 150 °C. Similarly, drop casting of PLA solution in chloroform (40 g/L) was performed to deposit a polymer layer onto the cellulose substrate surface, i.e., cellulose paper, cellulose nanopaper, and textile fabrics, prior to transferring into an oven at 120 °C with/without vacuum. After annealing, the samples were cooled down to room temperature in a desiccator. To uncover the irreversibly adsorbed Guiselin polymer layer, a rigorous solvent leaching process, i.e., changing fresh solvent minimum 6 times of 10 min each, was carried out to remove nonadsorbed polymers. In the above-mentioned cases, fresh toluene for removing the nonadsorbed PS and chloroform for leaching nonadsorbed PLA were applied, respectively. The samples were dried in a fume hood to remove the bulk solvent, and solvent residues were removed at room temperature in a vacuum oven with the pump on for 1 h prior to further characterization. The prolonged leaching step was performed for 9 days with daily changing fresh solvents twice per day.

### Surface Morphology by AFM

The surface topography of the representative films was collected through a Multimode 8 AFM instrument from Bruker AXS Inc. (Santa Barbara, CA, USA.) in air. Images were taken with a J scanner in tapping mode using NSC15/AIBS silicon cantilevers from MikroMasch (Tallinn, Estonia). A minimum of three images were taken per sample, and scans with at least 512 lines were performed over several portions of the films. Other than first-order polynomial flattening, no other image processing was carried out.

### Surface Chemical Composition by XPS

Surface elemental compositions of the cellulose substrates after solid-state adsorption were evaluated using an AXIS Ultra instrument (KratosAnalytical, U.K.). Samples were mounted on a linear sample holder with UHH compatible carbon tape and pre-evacuated overnight. A fresh piece of pure cellulosic filter paper (Whatman 1) was mounted and analyzed with each sample batch as an in situ ref (41). Measurements were performed using monochromated Al Kα irradiation at 100 W. Wide scans as well as high-resolution regions of C 1s were recorded on 3–4 locations for each sample, with a nominal analysis area of 400 × 800 μm^2^. Data analysis was performed using CasaXPS software package. Charge corrected wide scans were used for elemental analysis. Conditions in UHV remained satisfactory throughout the analysis. The low and stable contamination levels observed in the in situ reference sample, which was measured before and after each experiment, justified the analytical use of the C–C component in high-resolution C 1s spectra. In the XPS high-resolution C 1s spectra of cellulose nanopaper after solid-state adsorption, all the spectra were referenced to C–O at 286.7 eV.

### Thickness Evaluation of the Adsorbed Layer by Ellipsometry and X-ray Reflectometry

Ellipsometry was performed on a J.A. Woolam M2000UI (Lincoln, United States) spectroscopic ellipsometer (SE) with an auto retarder and rotating analyzer setup at incident angles of 60 and 70°. The thickness of the adsorbed layer was monitored by ellipsometry, where the spectroscopic technique measures the change of polarization and phase difference of p- and s-polarized light when light reflects from a surface. To reduce the number of free parameters during model fitting, the thickness of the oxide layer was determined before the deposition of any organic layer. A mapping scan with nine spots was conducted for each sample. The thickness of the deposited film after spin-coating and the solid-state adsorbed layer after solvent leaching was evaluated with a fixed value of oxide layer for each sample. The measurements were performed in the spectral range from 245 to 1690 nm wavelength. The data evaluation has been carried out using the manufactures’ software, CompleteEASE (version 6.51). The system of amorphous cellulose + PS Guiselin layer was modeled as a continuous film in a multilayer model, air/organic layer/SiO_*x*_/Si (substrate), by using a classic Cauchy model, which allowed for the thickness and refractive index determination. For the fitting of the real part of the refractive index *n*(λ), of the all the determined layers, a Cauchy model (eq 1) has been assumed; the imaginary part has been found to be negligible. Detailed model construction and comprehensive fitting explanation are provided in the Supporting Information.
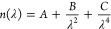
1where λ is the wavelength of radiation in micrometers. *A*, *B*, and *C* are the Cauchy coefficients, whereas all the coefficients are fitting as the positive value. For the substrate and silicon oxide, literature data have been used as listed in the software database.

The XRR measurements were performed with a SmartLab X-ray diffractometer set up using a 9 kW rotating anode, a germanium (Ge(220) × 2 double-bounce) monochromator (Cu Kα_1_ radiation, wavelength λ = 0.154 nm), soller slit, and a 10 mm divergence slit. The optics and samples were automatically aligned. Specular scans were taken by a symmetric variation of the incident and existing angles (θ) between 0 and 6° with a step width of and a velocity of 0.05 deg/min. In XRR, the layer thickness can be determined from the interference pattern, which consists of what are known as Kiessig fringes.^67^ The thickness of different layers, including SiO_*x*_, the cellulose substrate, and the adsorbed polymer layer was measured systematically to monitor the thickness change of the adsorbed polymer film. The substrate with thick silica is used because this results in high frequent oscillating signal in the reflectivity curves (Figure 2), which allow the facile distinction of very thin (nm range) polymer layers on top of it presenting much lower frequencies. The experimental XRR data was simulated employing a model independent approach (i.e., box with 32 slices) using the software package StochFit,^68^ which provides the possibility to fit the layer thickness and electron density profile using Parratt’s recursive formalism without any prior assumption.^69^ The electron density profile along the surface normal is generated to evaluate the density fluctuations and thickness changes before and after solid-state adsorption.

### Contact Angle

Contact angles of the modified cellulose surfaces with water (static, advancing, and receding) were measured using a Theta Flex optical tensiometer (Biolin Scientific, Sweden). A static water contact angle was recorded at 60 s after a sensile water drop was placed on the sample surface. A needle-in-drop sessile drop method was used for Quasi-static contact angles, i.e., advancing and receding contact angle, in attempt to evaluate the mobility of a drop on the surface in terms of contact-angle hysteresis (θ_a_–θ_r_).^70^ Contact angles were measured at two locations per substrate. At least triplicate samples were evaluated for each batch of samples. The reported average values and relative standard deviations were determined based on the above-mentioned six measurement points. The surface coverage of adsorbed polymer layer was calculated according to the Cassie–Baxter equation: cos θ_*m*_ = *X*_1_ cos θ_1_ + *X*_2_ cos θ_2_, where θ_*m*_ for measured contact angle, θ_1_ for measured full covered polymer static contact angle, θ_2_ for measured contact angle of pristine amorphous cellulose, and X_1_ and X_2_ for surface polymer fraction and surface fraction of amorphous cellulose, respectively.

### Characterizations of Cellulose Nanopaper after Solid-State Adsorption

Water vapor transmission rate (WVTR) of the pristine nanopaper and nanopaper with PLA Guiselin layer was measured using water vapor transmission rate tester Labthink W3/031(Labthink Instruments, Jinan, China). Dynamic water vapor sorption (DVS) of the nanopapers was characterized using DVS-1000 (Surface Measurement Systems, London, U.K.). The optical properties of transmittance and haze of the nanopapers were carried out using a Shimadzu Model UV-2600 system with an ISR-2600 Plus Integrating Sphere Attachment (Shimadzu, Japan), and the transmittance was measured between 900 and 200 nm. More detailed technical descriptions are described in the Supporting Information.

## Supporting Information Available

Details on preparations of each cellulosic substrates, data fitting model and process, characterizations of cellulose nanopapers after adsorption, and thickness evaluation with adsorption and followed by long-term solvent leaching (PDF)
Water repellency behavior of cellulose nanopaper with and without the PLA Guiselin layer (MOV)
Water repellence behavior of cellulose paper with and without the PLA Guiselin layer (MOV)
Water repellence behavior of cotton-based fabrics with and without the PLA Guiselin layer (MOV)

## Supplementary Material

am4c06182_si_001.pdf

am4c06182_si_002.mov

am4c06182_si_003.mov

am4c06182_si_004.mov

## References

[ref1] Wang Y.-K.; Jia F.; Li X.; Teale S.; Xia P.; Liu Y.; Chan P. T.-s.; Wan H.; Hassan Y.; Imran M.; et al. Self-assembled monolayer-based blue perovskite LEDs. Sci. Adv. 2023, 9 (36), eadh2140 10.1126/sciadv.adh2140. 37683007 PMC10491221

[ref2] Reimers J. R.; Ford M. J.; Marcuccio S. M.; Ulstrup J.; Hush N. S. Competition of van der Waals and chemical forces on gold-sulfur surfaces and nanoparticles. Nat. Rev. Chem 2017, 1 (2), 0017 10.1038/s41570-017-0017.

[ref3] Ansari M. Z.; Hussain I.; Mohapatra D.; Ansari S. A.; Rahighi R.; Nandi D. K.; Song W.; Kim S. H. Atomic layer deposition—a versatile toolbox for designing/engineering electrodes for advanced supercapacitors. Advanced Science 2024, 11 (1), 2303055 10.1002/advs.202303055. 37937382 PMC10767429

[ref4] Sun K.; Silveira O. J.; Ma Y.; Hasegawa Y.; Matsumoto M.; Kera S.; Krejčí O.; Foster A. S.; Kawai S. On-surface synthesis of disilabenzene-bridged covalent organic frameworks. Nat. Chem. 2023, 15 (1), 136–142. 10.1038/s41557-022-01071-3. 36344816 PMC9836936

[ref5] Li H.; Liebscher M.; Zhao D.; Yin B.; Du Y.; Yang J.; Kaliske M.; Mechtcherine V. A review of carbon fiber surface modification methods for tailor-made bond behavior with cementitious matrices. Prog. Mater. Sci. 2023, 132, 101040 10.1016/j.pmatsci.2022.101040.

[ref6] Guiselin O. Irreversible adsorption of a concentrated polymer solution. Europhys. Lett. 1992, 17 (3), 225–230. 10.1209/0295-5075/17/3/007.

[ref7] Fujii Y.; Yang Z.; Leach J.; Atarashi H.; Tanaka K.; Tsui O. K. Affinity of polystyrene films to hydrogen-passivated silicon and its relevance to the T g of the films. Macromolecules 2009, 42 (19), 7418–7422. 10.1021/ma901851w.

[ref8] Gin P.; Jiang N.; Liang C.; Taniguchi T.; Akgun B.; Satija S. K.; Endoh M. K.; Koga T. Revealed architectures of adsorbed polymer chains at solid-polymer melt interfaces. Phys. Rev. Lett. 2012, 109 (26), 265501 10.1103/PhysRevLett.109.265501. 23368578

[ref9] Napolitano S.; Wübbenhorst M. The lifetime of the deviations from bulk behaviour in polymers confined at the nanoscale. Nat. Commun. 2011, 2, 260 10.1038/ncomms1259.

[ref10] Kontturi K. S.; Solhi L.; Kontturi E.; Tammelin T. Adsorption of Polystyrene from Theta Condition on Cellulose and Silica Studied by Quartz Crystal Microbalance. Langmuir 2024, 40 (1), 568–579. 10.1021/acs.langmuir.3c02777. 38110337 PMC10786068

[ref11] Kontturi K. S.; Biegaj K.; Mautner A.; Woodward R. T.; Wilson B. P.; Johansson L. S.; Lee K. Y.; Heng J. Y. Y.; Bismarck A.; Kontturi E. Noncovalent Surface Modification of Cellulose Nanopapers by Adsorption of Polymers from Aprotic Solvents. Langmuir 2017, 33 (23), 5707–5712. 10.1021/acs.langmuir.7b01236. 28520438

[ref12] Song Z.; Rodríguez-Tinoco C.; Mathew A.; Napolitano S. Fast equilibration mechanisms in disordered materials mediated by slow liquid dynamics. Sci. Adv. 2022, 8 (15), eabm7154 10.1126/sciadv.abm7154. 35427165 PMC9012462

[ref13] Ren W.; Wang X.; Shi J.; Xu J.; Taneda H.; Yamada N. L.; Kawaguchi D.; Tanaka K.; Wang X. The role of the molecular weight of the adsorbed layer on a substrate in the suppressed dynamics of supported thin polystyrene films. Soft Matter 2022, 18 (10), 1997–2005. 10.1039/D2SM00067A. 35195149

[ref14] Napolitano S. Irreversible adsorption of polymer melts and nanoconfinement effects. Soft Matter 2020, 16 (23), 5348–5365. 10.1039/D0SM00361A. 32419002

[ref15] Ren W.; Hong Y.; Wei H.; Xu J.; Zhang C.; Zhou X.; Wang X. Structure of the Poly (methyl methacrylate) Adsorbed Layer Determined by the Surface Chemistry of the Substrate. Macromolecules 2023, 56 (4), 1410–1418. 10.1021/acs.macromol.2c02169.

[ref16] Tian H.; Bi C.; Li Z.; Wang C.; Zuo B. Metastable polymer adsorption dictates the dynamical gradients at interfaces. Macromolecules 2023, 56 (11), 4346–4353. 10.1021/acs.macromol.3c00414.

[ref17] McCrum N. G.; Buckley C. P.; Bucknall C. B. Principles of Polymer Engineering; Oxford University Press, 1997; pp 84–116.

[ref18] Heise K.; Kontturi E.; Allahverdiyeva Y.; Tammelin T.; Linder M. B.; Ikkala O.; Ikkala O. Nanocellulose: recent fundamental advances and emerging biological and biomimicking applications. Adv. Mater. 2021, 33 (3), 2004349 10.1002/adma.202004349. PMC11468234 33289188

[ref19] De France K.; Zeng Z.; Wu T.; Nyström G. Functional materials from nanocellulose: utilizing structure-property relationships in bottom-up fabrication. Adv. Mater. 2021, 33 (28), 2000657 10.1002/adma.202000657. PMC11468739 32267033

[ref20] Wågberg L.; Erlandsson J. The use of layer-by-layer self-assembly and nanocellulose to prepare advanced functional materials. Adv. Mater. 2021, 33 (28), 2001474 10.1002/adma.202001474. PMC11468756 32767441

[ref21] Yang X.; Biswas S. K.; Han J.; Tanpichai S.; Li M. C.; Chen C.; Zhu S.; Das A. K.; Yano H. Surface and interface engineering for nanocellulosic advanced materials. Adv. Mater. 2021, 33 (28), 2002264 10.1002/adma.202002264. PMC11468146 32902018

[ref22] Frka-Petesic B.; Parton T. G.; Honorato-Rios C.; Narkevicius A.; Ballu K.; Shen Q.; Lu Z.; Ogawa Y.; Haataja J. S.; Droguet B. E.; et al. Structural color from cellulose nanocrystals or chitin nanocrystals: self-assembly, optics, and applications. Chem. Rev. 2023, 123 (23), 12595–12756. 10.1021/acs.chemrev.2c00836. 38011110 PMC10729353

[ref23] Ferreira E. S.; Rezende C. A.; Cranston E. D. Fundamentals of cellulose lightweight materials: bio-based assemblies with tailored properties. Green Chem. 2021, 23 (10), 3542–3568. 10.1039/D1GC00326G.

[ref24] Etale A.; Onyianta A. J.; Turner S. R.; Eichhorn S. J. Cellulose: a review of water interactions, applications in composites, and water treatment. Chem. Rev. 2023, 123 (5), 2016–2048. 10.1021/acs.chemrev.2c00477. 36622272 PMC9999429

[ref25] Solhi L.; Guccini V.; Heise K.; Solala I.; Niinivaara E.; Xu W.; Mihhels K.; Kröger M.; Meng Z.; Wohlert J.; et al. Understanding Nanocellulose-Water Interactions: Turning a Detriment into an Asset. Chem. Rev. 2023, 123 (5), 1925–2015. 10.1021/acs.chemrev.2c00611. 36724185 PMC9999435

[ref26] Niinivaara E.; Faustini M.; Tammelin T.; Kontturi E. Water vapor uptake of ultrathin films of biologically derived nanocrystals: quantitative assessment with quartz crystal microbalance and spectroscopic ellipsometry. Langmuir 2015, 31 (44), 12170–12176. 10.1021/acs.langmuir.5b01763. 26461931

[ref27] Oksman K.; Aitomäki Y.; Mathew A. P.; Siqueira G.; Zhou Q.; Butylina S.; Tanpichai S.; Zhou X.; Hooshmand S. Review of the recent developments in cellulose nanocomposite processing. Composites, Part A 2016, 83, 2–18. 10.1016/j.compositesa.2015.10.041.

[ref28] Geng S.; Wloch D.; Herrera N.; Oksman K. Large-scale manufacturing of ultra-strong, strain-responsive poly (lactic acid)-based nanocomposites reinforced with cellulose nanocrystals. Compos. Sci. Technol. 2020, 194, 108144 10.1016/j.compscitech.2020.108144.

[ref29] Ghasemlou M.; Daver F.; Ivanova E. P.; Habibi Y.; Adhikari B. Surface modifications of nanocellulose: From synthesis to high-performance nanocomposites. Prog. Polym. Sci. 2021, 119, 101418 10.1016/j.progpolymsci.2021.101418.

[ref30] Thomas B.; Raj M. C.; Joy J.; Moores A.; Joy J.; Moores A.; Drisko G. L.; Sanchez C. Nanocellulose, a versatile green platform: from biosources to materials and their applications. Chem. Rev. 2018, 118 (24), 11575–11625. 10.1021/acs.chemrev.7b00627. 30403346

[ref31] Beaumont M.; Tardy B. L.; Reyes G.; Koso T. V.; Schaubmayr E.; Jusner P.; King A. W.; Dagastine R. R.; Potthast A.; Rojas O. J.; et al. Assembling native elementary cellulose nanofibrils via a reversible and regioselective surface functionalization. J. Am. Chem. Soc. 2021, 143 (41), 17040–17046. 10.1021/jacs.1c06502. 34617737 PMC8532154

[ref32] Mujtaba M.; Negi A.; King A. W.; Zare M.; Kuncova-Kallio J. Surface modifications of nanocellulose for drug delivery applications; a critical review. Curr. Opin. Biomed. Eng. 2023, 28, 100475 10.1016/j.cobme.2023.100475.

[ref33] Mautner A.; Lee K.-Y.; Lahtinen P.; Hakalahti M.; Tammelin T.; Li K.; Bismarck A. Nanopapers for organic solvent nanofiltration. Chem. Commun. 2014, 50 (43), 5778–5781. 10.1039/C4CC00467A. 24752201

[ref34] Khakalo A.; Mäkelä T.; Johansson L.-S.; Orelma H.; Tammelin T. High-throughput tailoring of nanocellulose films: From complex bio-based materials to defined multifunctional architectures. ACS Appl. Bio Mater. 2020, 3 (11), 7428–7438. 10.1021/acsabm.0c00576. PMC7673207 33225237

[ref35] Durning C.; O’shaughness B.; Sawhney U.; Nguyen D.; Majewski J.; Smith G. Adsorption of poly (methyl methacrylate) melts on quartz. Macromolecules 1999, 32 (20), 6772–6781. 10.1021/ma981785k.

[ref36] Fujii Y.; Yang Z.; Leach J.; Atarashi H.; Tanaka K.; Tsui O. K. C. Affinity of Polystyrene Films to Hydrogen-Passivated Silicon and Its Relevance to theTgof the Films. Macromolecules 2009, 42 (19), 7418–7422. 10.1021/ma901851w.

[ref37] Vanroy B.; Wübbenhorst M.; Napolitano S. Crystallization of thin polymer layers confined between two adsorbing walls. ACS Macro Lett. 2013, 2 (2), 168–172. 10.1021/mz300641x. 35581781

[ref38] Bal J. K.; Beuvier T.; Unni A. B.; Chavez Panduro E. A.; Vignaud G.; Delorme N.; Chebil M. S.; Grohens Y.; Gibaud A. Stability of polymer ultrathin films (< 7 nm) made by a top-down approach. ACS Nano 2015, 9 (8), 8184–8193. 10.1021/acsnano.5b02381. 26149069

[ref39] Napolitano S.; Pilleri A.; Rolla P.; Wubbenhorst M. Unusual deviations from bulk behavior in ultrathin films of poly (tert-butylstyrene): Can dead layers induce a reduction of T g?. ACS Nano 2010, 4 (2), 841–848. 10.1021/nn9014517. 20112970

[ref40] Xu W.; Mihhels K.; Kotov N.; Lepikko S.; Ras R. H.; Johnson C. M.; Pettersson T.; Kontturi E. Solid-state polymer adsorption for surface modification: The role of molecular weight. J. Colloid Interface Sci. 2022, 605, 441–450. 10.1016/j.jcis.2021.07.062. 34333417

[ref41] Johansson L. S.; Campbell J. Reproducible XPS on biopolymers: cellulose studies. Surf. Interface Anal. 2004, 36 (8), 1018–1022. 10.1002/sia.1827.

[ref42] Simavilla D. N.; Huang W.; Vandestrick P.; Ryckaert J.-P.; Sferrazza M.; Napolitano S. Mechanisms of Polymer Adsorption onto Solid Substrates. ACS Macro Lett. 2017, 6 (9), 975–979. 10.1021/acsmacrolett.7b00473. 35650878

[ref43] Ligoure C.; Leibler L. Thermodynamics and kinetics of grafting end-functionalized polymers to an interface. J. Phys. 1990, 51 (12), 1313–1328. 10.1051/jphys:0199000510120131300.

[ref44] Ehmann H. M. A.; Werzer O.; Pachmajer S.; Mohan T.; Amenitsch H.; Resel R.; Kornherr A.; Stana-Kleinschek K.; Kontturi E.; Spirk S. Surface-Sensitive Approach to Interpreting Supramolecular Rearrangements in Cellulose by Synchrotron Grazing Incidence Small-Angle X-ray Scattering. ACS Macro Lett. 2015, 4 (7), 713–716. 10.1021/acsmacrolett.5b00306. 35596493

[ref45] Gin P.; Jiang N.; Liang C.; Taniguchi T.; Akgun B.; Satija S. K.; Endoh M. K.; Koga T. Revealed architectures of adsorbed polymer chains at solid-polymer melt interfaces. Phys. Rev. Lett. 2012, 109 (26), 265501 10.1103/PhysRevLett.109.265501. 23368578

[ref46] Kim J.; Montero G.; Habibi Y.; Hinestroza J. P.; Genzer J.; Argyropoulos D. S.; Rojas O. J. Dispersion of cellulose crystallites by nonionic surfactants in a hydrophobic polymer matrix. Polym. Eng. Sci. 2009, 49 (10), 2054–2061. 10.1002/pen.21417.

[ref47] Carelli C.; Young R.; Jones R. A. L.; Sferrazza M. The contrast match neutron reflectivity technique for the study of broad polymer/polymer interfaces. Nucl. Instrum. Methods Phys. Res., Sect. B 2006, 248 (1), 170–174. 10.1016/j.nimb.2006.04.067.

[ref48] Kontturi E.; Thüne P. C.; Niemantsverdriet J. Cellulose model surfaces simplified preparation by spin coating and characterization by X-ray photoelectron spectroscopy, infrared spectroscopy, and atomic force microscopy. Langmuir 2003, 19 (14), 5735–5741. 10.1021/la0340394.

[ref49] Ni S. Poly (L-Lactic Acid) Langmuir Monolayers at the Air/Water Interface and Langmuir-Blodgett Films on Solid Substrates: Phase Behavior, Surface Morphology, and Crystallinity; Virginia Tech, 2006; .

[ref50] Fleer G.; Stuart M. C.; Scheutjens J. M.; Cosgrove T.; Vincent B. Polymers at Interfaces; Springer Science & Business Media, 1993; pp 27–42.

[ref51] Chinga-Carrasco G.; Tobjörk D.; Österbacka R. Inkjet-printed silver nanoparticles on nano-engineered cellulose films for electrically conducting structures and organic transistors: concept and challenges. J. Nanopart. Res. 2012, 14, 1213 10.1007/s11051-012-1213-x.

[ref52] Costa S. V.; Pingel P.; Janietz S.; Nogueira A. F. Inverted organic solar cells using nanocellulose as substrate. J. Appl. Polym. Sci. 2016, 133 (28), 43679 10.1002/app.43679.

[ref53] Habibi Y. Key advances in the chemical modification of nanocelluloses. Chem. Soc. Rev. 2014, 43 (5), 1519–1542. 10.1039/C3CS60204D. 24316693

[ref54] Heux L.; Chauve G.; Bonini C. Nonflocculating and chiral-nematic self-ordering of cellulose microcrystals suspensions in nonpolar solvents. Langmuir 2000, 16 (21), 8210–8212. 10.1021/la9913957.

[ref55] Shimizu M.; Saito T.; Isogai A. Bulky quaternary alkylammonium counterions enhance the nanodispersibility of 2, 2, 6, 6-tetramethylpiperidine-1-oxyl-oxidized cellulose in diverse solvents. Biomacromolecules 2014, 15 (5), 1904–1909. 10.1021/bm500384d. 24750066

[ref56] Vuoriluoto M.; Orelma H.; Johansson L.-S.; Zhu B.; Poutanen M.; Walther A.; Laine J.; Rojas O. J. Effect of Molecular Architecture of PDMAEMA-POEGMA Random and Block Copolymers on Their Adsorption on Regenerated and Anionic Nanocelluloses and Evidence of Interfacial Water Expulsion. J. Phys. Chem. B 2015, 119 (49), 15275–15286. 10.1021/acs.jpcb.5b07628. 26560798

[ref57] Utsel S.; Bruce C.; Pettersson T. r.; Fogelström L.; Carlmark A.; Malmström E.; Wågberg L. Physical tuning of cellulose-polymer interactions utilizing cationic block copolymers based on PCL and quaternized PDMAEMA. ACS Appl. Mater. Interfaces 2012, 4 (12), 6796–6807. 10.1021/am301981r. 23157287

[ref58] Utsel S.; Carlmark A.; Pettersson T.; Bergström M.; Malmström E. E.; Wågberg L. Synthesis, adsorption and adhesive properties of a cationic amphiphilic block copolymer for use as compatibilizer in composites. Eur. Polym. J. 2012, 48 (7), 1195–1204. 10.1016/j.eurpolymj.2012.05.004.

[ref59] Salajková M.; Berglund L. A.; Zhou Q. Hydrophobic cellulose nanocrystals modified with quaternary ammonium salts. J. Mater. Chem. 2012, 22 (37), 19798–19805. 10.1039/c2jm34355j.

[ref60] Zhou Q.; Greffe L.; Baumann M. J.; Malmström E.; Teeri T. T.; Brumer H. Use of xyloglucan as a molecular anchor for the elaboration of polymers from cellulose surfaces: a general route for the design of biocomposites. Macromolecules 2005, 38 (9), 3547–3549. 10.1021/ma047712k.

[ref61] Zhou Q.; Brumer H.; Teeri T. T. Self-organization of cellulose nanocrystals adsorbed with xyloglucan oligosaccharide- poly (ethylene glycol)- polystyrene triblock copolymer. Macromolecules 2009, 42 (15), 5430–5432. 10.1021/ma901175j.

[ref62] Hatton F. L.; Ruda M.; Lansalot M.; D’Agosto F.; Malmstrom E.; Carlmark A. Xyloglucan-functional latex particles via RAFT-mediated emulsion polymerization for the biomimetic modification of cellulose. Biomacromolecules 2016, 17 (4), 1414–1424. 10.1021/acs.biomac.6b00036. 26913868

[ref63] Cunha A. G.; Zhou Q.; Larsson P. T.; Berglund L. A. Topochemical acetylation of cellulose nanopaper structures for biocomposites: mechanisms for reduced water vapour sorption. Cellulose 2014, 21, 2773–2787. 10.1007/s10570-014-0334-z.

[ref64] Cunha A. G.; Lundahl M.; Ansari M. F.; Johansson L.-S.; Campbell J. M.; Rojas O. J. Surface structuring and water interactions of nanocellulose filaments modified with organosilanes toward wearable materials. ACS Appl. Nano Mater. 2018, 1 (9), 5279–5288. 10.1021/acsanm.8b01268. 30320301 PMC6167725

[ref65] Ranjan V. P.; Joseph A.; Goel S. Microplastics and other harmful substances released from disposable paper cups into hot water. J. Hazard. Mater. 2021, 404, 124118 10.1016/j.jhazmat.2020.124118. 33091697

[ref66] Eronen P.; Laine J.; Ruokolainen J.; Österberg M. Comparison of multilayer formation between different cellulose nanofibrils and cationic polymers. J. Colloid Interface Sci. 2012, 373 (1), 84–93. 10.1016/j.jcis.2011.09.028. 21993549

[ref67] Kiessig H. Interference of X-rays in thick layers. Ann. Phys. 1931, 10, 193.

[ref68] Werzer O.; Resel R. Model-independent X-ray reflectivity fitting for structure analysis of poly (3-hexylthiophene) films. Macromolecules 2013, 46 (9), 3529–3533. 10.1021/ma400147g.

[ref69] Parratt L. G. Surface Studies of Solids by Total Reflection of X-Rays. Phys. Rev. 1954, 95 (2), 359–369. 10.1103/physrev.95.359.

[ref70] Huhtamäki T.; Tian X.; Korhonen J. T.; Ras R. H. Surface-wetting characterization using contact-angle measurements. Nat. Protoc. 2018, 13 (7), 1521–1538. 10.1038/s41596-018-0003-z. 29988109

