# Supplementary material for: Interfacial Engineering of Soft Matter Substrates by Solid-State Polymer Adsorption
Source: ACS Appl Mater Interfaces. 2024 Jun 12;16(25):32874–85. doi: 10.1021/acsami.4c06182 (PMC11212027; doi:10.1021/acsami.4c06182)
Supplement: Supplementary file 1 — am4c06182_si_001.pdf [file am4c06182_si_001.pdf]

Supporting Information for

Interfacial engineering of soft matter substrates by solid-state polymer adsorption

Wenyang Xu,[1,2,3,*] Oliver Werzer,[4] Panagiotis Spiliopoulos,[1] Karl Mihhels,[1] Qixiang Jiang,[5] Zhuojun Meng,[1] Han Tao,[1] Roland Resel,[6] Tekla Tammelin,[7] Torbjörn Pettersson,[2,8] Eero Kontturi[1,*]

[1] Department of Bioproducts and Biosystems, School of Chemical Engineering, Aalto University, P.O. Box 16300, FI-00076 Aalto, Finland

[2] Department of Fibre and Polymer Technology, KTH Royal Institute of Technology, Teknikringen 56, SE-10044 Stockholm, Sweden

[3] Laboratory of Natural Materials Technology, Åbo Akademi University, FI-20500 Turku, Finland

[4] Joanneum Research, Institute for Sensors, Photonics and Manufacturing Technologies, Franz-Pichler-Strasse 30, 8160 Weiz, Austria

[5] Polymer and Composite Engineering (PaCE) Group, Institute of Materials Chemistry, Faculty of Chemistry, University of Vienna, Währinger Straße 42, A-1090 Vienna, Austria

[6] Institute of Solid State Physics, NAWI Graz, Graz University of Technology, Petersgasse 16, 8010 Graz, Austria

[7] Biomass processing and products, VTT Technical Research Centre of Finland Ltd., FI-02044 Espoo, Finland

[8] Wallenberg Wood Science Centre, KTH Royal Institute of Technology, Teknikringen 56, SE-10044 Stockholm, Sweden

*To whom correspondence should be addressed to: Department of Bioproducts and Biosystems, School of Chemical Engineering, Aalto University, P.O. Box 16300, FI-00076 Aalto, Finland. E-mail: Eero.Kontturi@aalto.fi; Wenyang.2.Xu@aalto.fi

This PDF file includes:

Supporting text
Figures S1 to S12
Tables S1 to S2
Legends for Movies S1 to S3
SI References

Other supporting materials for this manuscript include the following:

Movies S1 to S3

Supporting Information Text

Experimental procedures

Preparation of cellulose nanocrystals (CNCs)

CNCs were prepared from cotton based Whatman 1 filter paper using a commonly used protocol with 64 wt.% sulfuric acid hydrolysis. Whatman filter paper (10 g) was finely grinded and hydrolysed in 175 mL 64 wt.% at 45 °C for 45 min.[1] Hydrolysis was quenched by the addition of 10-fold Milli-Q water. The suspension was then centrifuged at 10000 rpm for 15-30 min removing excess water. Fresh water was then replenished twice to dilute and remove residual acids. The resulting CNCs was further dialyzed against Milli-Q water until the conductivity of the dialysis water was below 5 µS/cm.[3] The dialyzed CNC dispersion was then counterion exchanged by adding 0.1 M sodium hydroxide until the pH value reaches 7. In order to remove surface impurities, the CNCs dispersion was freeze-dried followed by Soxhlet extraction with ethanol for 48 h, according to a modified method from Labet and Thielemans.[2]

Preparation of cellulose nanofibrils (CNFs)

CNFs were produced from once-dried bleached birch kraft pulp (13% glucose, 23% xylose and 0.15% methyl glucuronic acid) from a Finnish pulp mill by mechanical disintegration according to the procedure described in Mäkelä et al.[3] Briefly, the chemical pulp at 1.7 wt.% consistency was first dispersed using a high shear Diaf dissolver (Minibatch Type20) for 10 min at 700 rpm followed by pre-refining (2 cycles) in a Masuko grinder (Supermasscolloider MKZA 10-15J, Masuko Sangyo Co., Japan) at 1500 rpm. The final nanofibrillation was carried out using Microfluidizer (Microfluidics M-7115-30 Microfluidics Corp., USA) where the pre-refined pulp was fluidized 7 times through the chambers (the first pass at 50 bar and six passes at the operating pressure of 1800 bar). CNF appears as a viscous gel with a final solid content of 1.6 wt.% with the yield stress of ~55 Pa and apparent viscosity values of ~179 000 mPas at 0.5 rpm and ~18 000 mPas at 10 rpm measured with a Brookfield rheometer RVDV with spindle type V73 at 0.8 wt.% solid content.

CNF film preparation

CNF film production is based on cast-coating of the CNF gel (plasticized with sorbitol, 30 wt.% solids in dry CNF film from Sigma Aldrich) on a supporting plastic substrate with precise control of adhesion, spreading and drying of CNF layer using a semi-pilot scale roll-to-roll converting unit (Coatema, Germany) as described in Pöhler et al[4] and Mäkelä et al[34]. Spreading and adhesion were controlled by plasma activation (Ar-plasma unit MKII 2000 W m^{-2} h^{-1}) using Vetaphone Corona-Plus, Denmark to adjust the surface energy of the plastic substrate. After drying via evaporation, the CNF film is separated from the plastic substrate and cut into self-standing A4 sheets.

Trimethylsilyl cellulose (TMSC) preparation

TMSC was synthesized and characterized according to a method described in detail elsewhere.[56] In brief, the cellulose powder (1 g) was dissolved in dimethylacetamide with LiCl (8 g LiCl/ 100 mL DMAc) at 80 °C in a water bath. After the cellulose had completely dissolved, 10 mL of HMDS was added in a steady flow within 1 h under a nitrogen atmosphere. The mixture was cooled for TMSC crystallization. The crystallized TMSC was filtered and dissolved into 40 mL of tetrahydrofuran and recrystallized in 600 mL of methanol. After filtration, the recrystallized TMSC was washed several times with methanol and dried in a vacuum desiccator.

Ellipsometry model and fitting details

The used model Si/SiO_x/Cellulose and the adsorbed PS layer/Air for ellipsometry fitting is listed as following: the cellulose substrate and the adsorbed PS with cellulose are considered as one united film during analyses. Cauchy model was applied to fit the data, where fit variables are 'thickness', 'A', and 'B'. All the parameters are fitting as positive values. The SiO_x layer was measured before thin film deposition. The Cauchy model with variables of thickness and 'A' is applied. The fitted values are fixed for the top layer fitting. The Si layer with fixed variables of tabulated optical constants (n and k) values is used according to the software. The good fit in **Figure S3 and S8** of the model to the data validates the use of the model described above. In other words, the model structure and model assumptions are correct, and refractive index follows Cauchy dispersion equation. Thus, the measured thickness represents the sample well.

Water vapour transmission rate

Water vapour transmission rate (WVTR) of the nanocellulose films was measured using water vapour transmission rate tester Labthink W3/031(Labthink Instruments, Jinan, China). The nanocellulose films were laminated between two aluminium foils (Aluminium masks with hole, Ametek). The hole in the centre of the foil allowed the nanocellulose film for a testing area of 4.9 cm^2. Ultrapure water was used for the test. The water was filled in a sample holder, on top of which the laminated film was placed. The sample holders were placed in the WVTR tester. The nanocellulose films were preconditioned for 1 h and, subsequently tested at 38°C and a RH of 90%.

Dynamic vapour sorption

Dynamic water vapour sorption (DVS) by pristine nanopaper and nanopaper with PLA Guiselin layer was characterized using DVS-1000 (Surface Measurement Systems, London, U.K.). The water vapour uptake was recorded at 25 °C by monitoring the change in mass as the sample was exposed to 0–95–0% RH cycle in steps (0, 5, 15,25, 35, 45, 55, 65, 75, 85, 95% RH) for 3 h at each step. Sample size of around 15 mg and the flow rate of 100 sccm were applied.

Ultraviolet–visible light (UV-vis) spectroscopy

The optical properties of transmittance and haze of the nanopapers were carried out using a Shimadzu Model UV-2600 system with an ISR-2600 Plus Integrating Sphere Attachment (Shimadzu, Japan), and the transmittance was measured between 900 nm and 200 nm. Optical haze was used to quantify the percentage of the forward light scattering as follows: $Haze(\%) = (\frac{T_4}{T_2} - \frac{T_3}{T_1}) \times 100$, where T_1, T_2, T_3 and T_4 are defined as the background checking, total transmitted illumination, beam checking, and pure diffusive transmittances, respectively.[6]

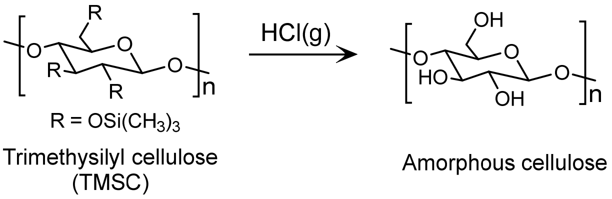

Figure S1. The mechanism of regeneration of amorphous cellulose by the hydrolysis of trimethylsilyl cellulose (TMSC) *via* HCl vapor.

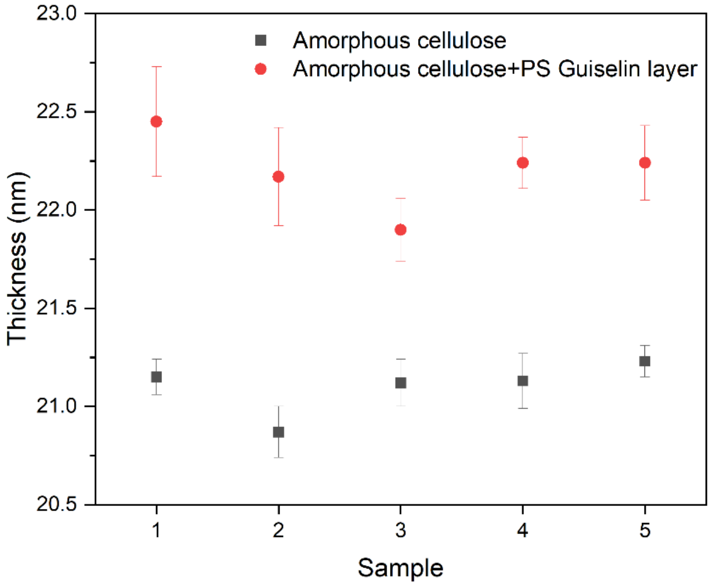

Figure S2. Thickness evaluation of different amorphous cellulose thin films before and after deposition of polystyrene Guiselin layer *via* solid-state adsorption using spectroscopic ellipsometer. The error value was calculated based on 9 different points of the same sample.

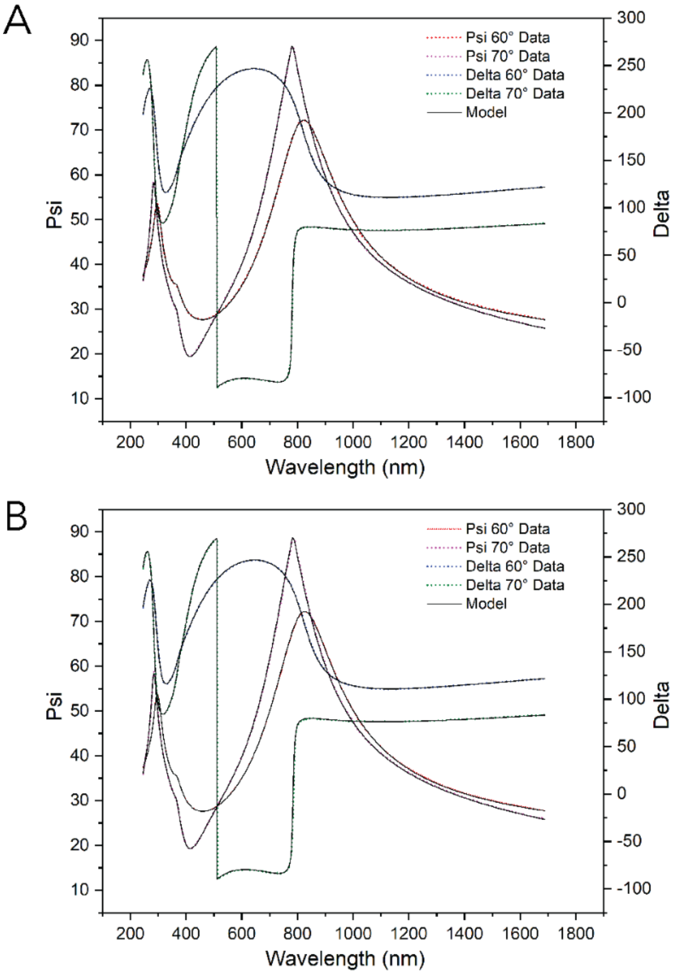

Figure S3. Ellipsometry raw data and fitting curve of representative samples from (A) amorphous cellulose and (B) amorphous cellulose with PS Guiselin layer. A comprehensive explanation on model construction and data analyses were referred to above section of '*Ellipsometry model and fitting details*'.

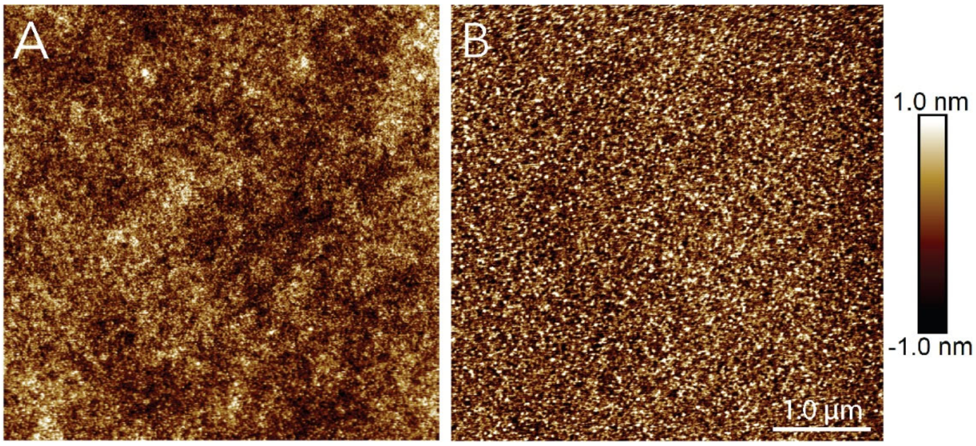

Figure S4. AFM topographies of (A) spin-coated TMSC film (R_q=0.35 nm) and (B) regenerated amorphous cellulose model film (R_q=0.45 nm).

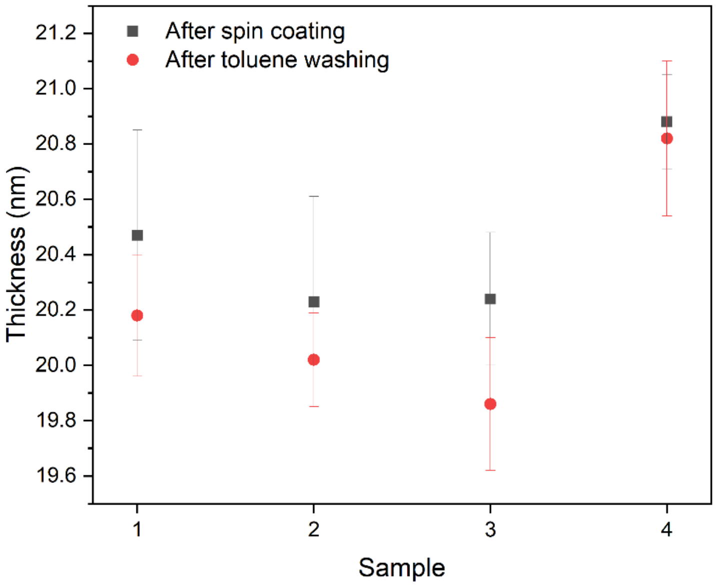

Figure S5. Thickness comparison of amorphous cellulose films after regeneration (*i.e.*, before toluene washing) and after toluene washings determined by spectroscopic ellipsometer.

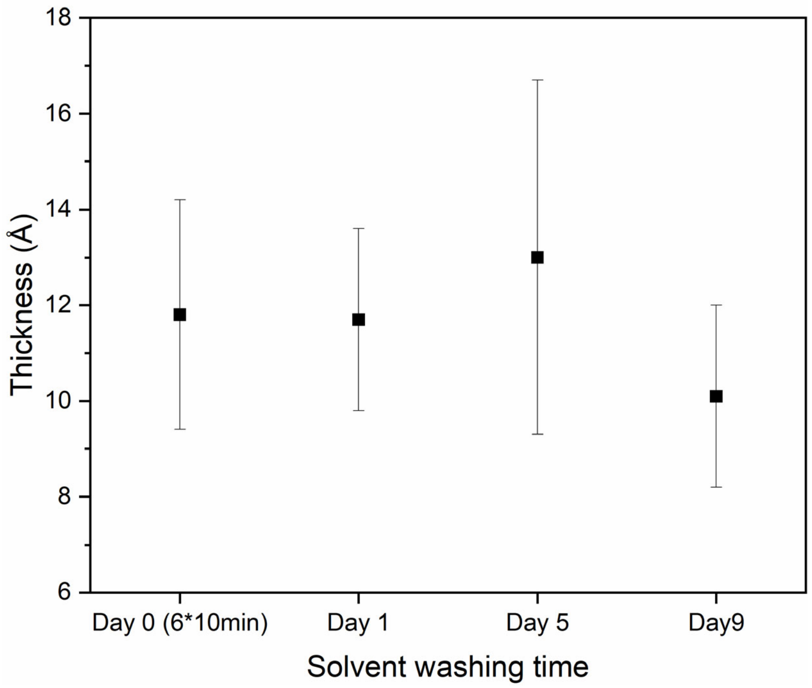

Figure S6. Thickness change of the prolonged solvent leaching time for amorphous cellulose together with PS560k Guiselin layer, as revealed spectroscopic ellipsometer.

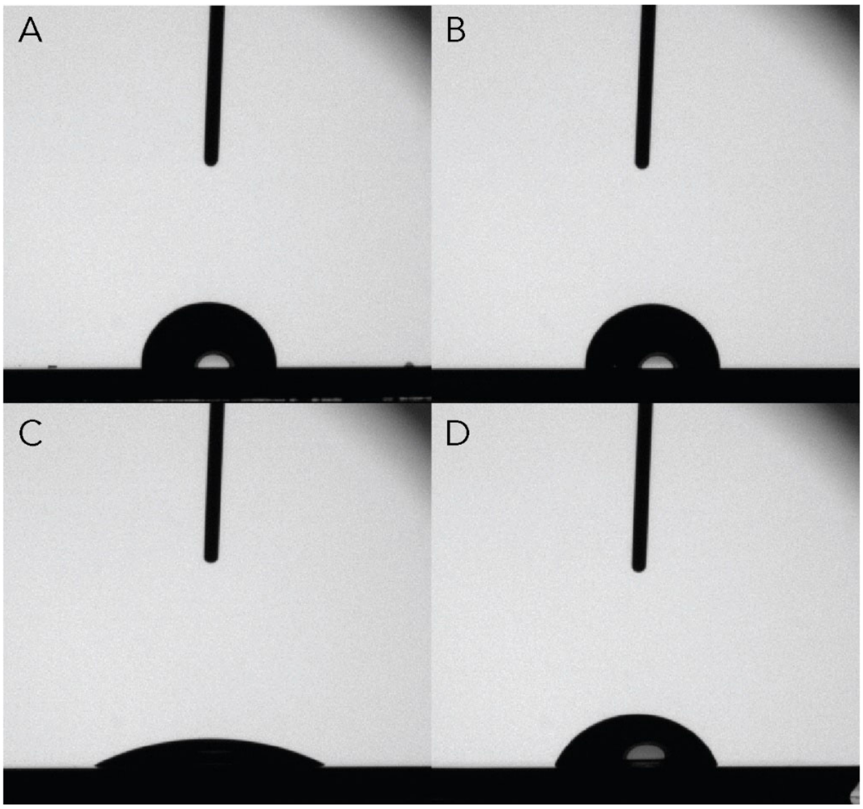

Figure S7. The static contact angle of the representative samples of (A) spin-coated PS560k, (B) PS560k Guiselin layer on silica, (C) amorphous cellulose, and (D) amorphous cellulose with PS560k Guiselin layer.

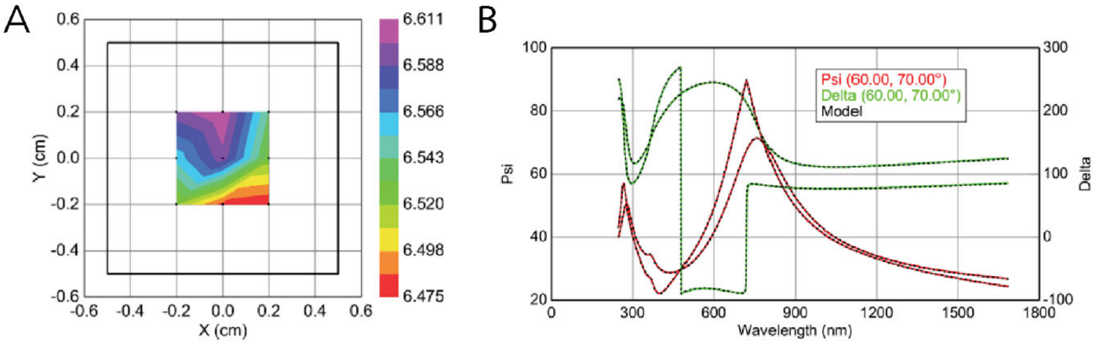

Figure S8. Thickness evaluation using spectroscopic ellipsometer. (A) Thickness mapping versus measurement position for representative sample of PLA Guiselin layer on planar silica surface. (B) Data fitting at position (X=0, and Y=0) of PLA Guiselin layer on silica surface.

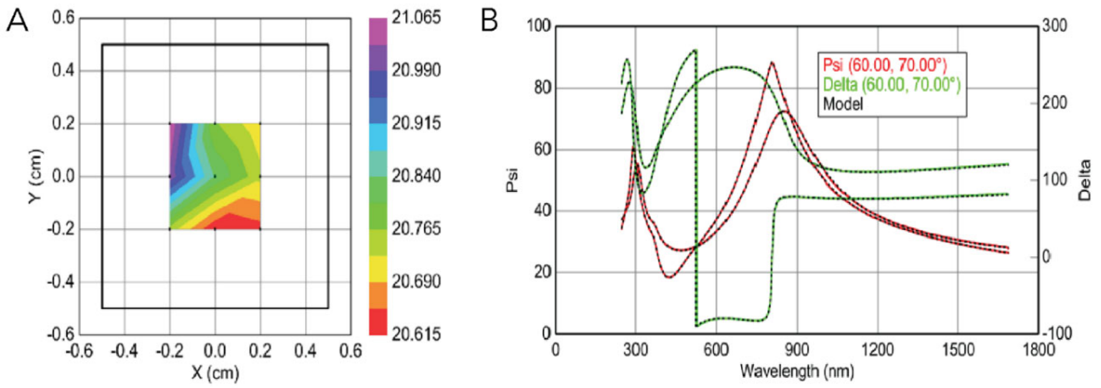

Figure S9. Thickness evaluation using spectroscopic ellipsometer. (A) Thickness evaluation of PLA Guiselin layer on amorphous cellulose with thickness mapping versus measurement position for representative sample 1 in **Figure 3C**. (B) Data fitting at position (X=0, and Y=0) of representative sample 1 in **Figure 3C**.

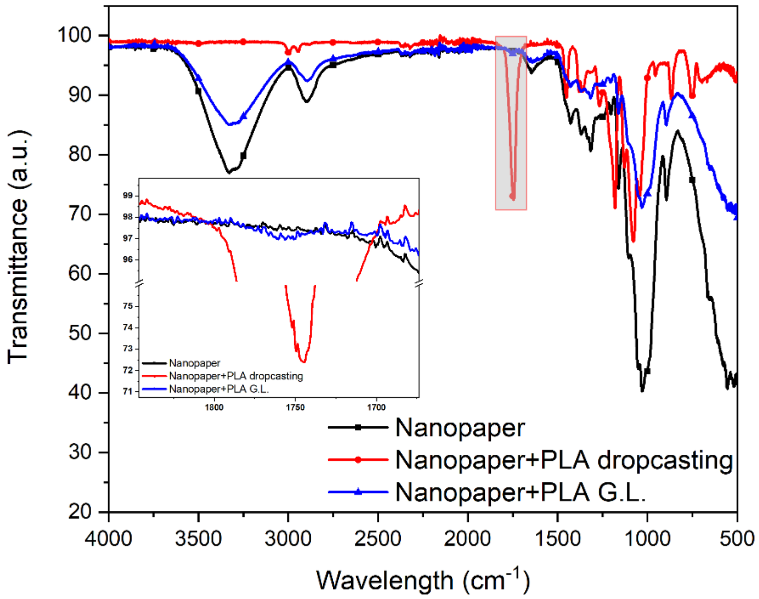

Figure S10. ATR-FTIR spectra of the cellulose nanopaper substrate before and after drop casting of PLA, and cellulose nanopaper with PLA Guiselin layer.

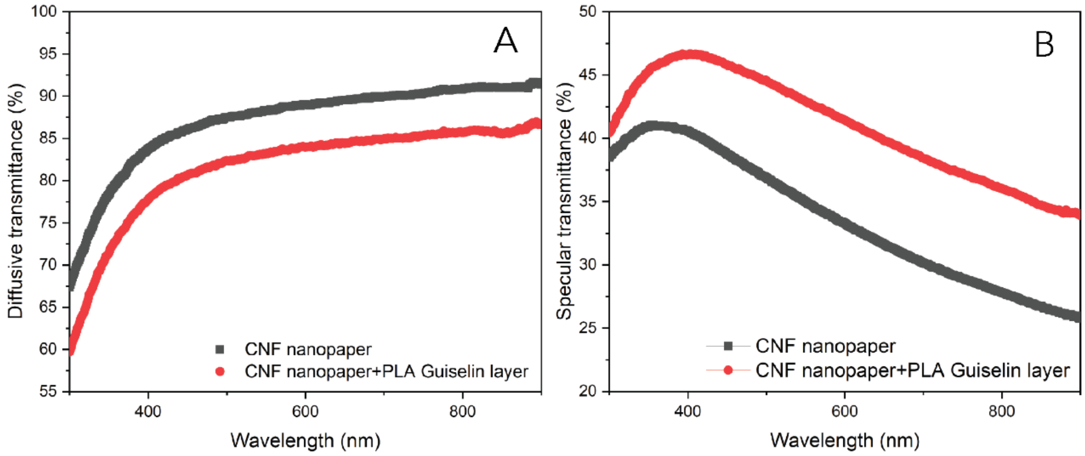

Figure S11. Optical properties of nanopaper. (A)Diffusive transmittance and (B) specular transmittance of the cellulose nanopaper before and after solid-state adsorption of PLA.

Table S1. Roughness and water contact angles (static, advancing (θ_a), and receding (θ_r) contact angles) of cellulose nanopaper before and after PLA Guiselin layer formation.

Samples	Roughness (Rq)*/nm	Static contact angle /°	θ_a, θ_r /°
Cellulose nanopaper	21.7	24.3±0.6(t=0s)<br>14.7±0.3(t=60s)	-
Cellulose nanopaper+PLA Guiselin layer	15.1	81.7±0.2(t=0s)<br>81.4±0.1(t=60s)	90.4±3.3,<br>80.7±1.9

*Root mean square roughness (R_q) is obtained from AFM height image. – quasi-static contact angle for cellulose nanopaper before PLA adsorption was not measured due to water adhesion interfering contact angel measurements; The practical performance of water repellence of cellulose nanopaper can be found in **Figure 5** and **Video S1** (separate file). The advancing and receding contact angle of pristine cellulose nanopaper could not be measured effectively and accurately due to its hygroscopic nature.

Table S2. Haze and water vapor transmission rate of cellulose nanopaper before and after solid-state adsorption of PLA.

	Cellulose nanopaper	Cellulose nanopaper after annealing	Cellulose nanopaper + PLA Guiselin layer.
Haze (at 550 nm)	0.39	0.48	0.51
Water vapor transmission rate (g/m^2 24h)	2660±177	-	2458±183

Movie S1 (separate file). Video S1. Water repellence behavior of cellulose nanopaper with and without PLA Guiselin layer. **Video S2.** Water repellence behavior of cellulose paper with and without PLA Guiselin layer. **Video S3.** Water repellence behavior of cotton-based fabrics with and without PLA Guiselin layer.

SI References

(1) Dong, X. M.; Revol, J.-F.; Gray, D. G. Effect of microcrystallite preparation conditions on the formation of colloid crystals of cellulose. *Cellulose* **1998**, *5* (1), 19-32.
(2) Labet, M.; Thielemans, W. Improving the reproducibility of chemical reactions on the surface of cellulose nanocrystals: ROP of ε-caprolactone as a case study. *Cellulose* **2011**, *18* (3), 607-617.
(3) Mäkelä, T.; Kainlauri, M.; Willberg-Keyriläinen, P.; Tammelin, T.; Forsström, U. Fabrication of micropillars on nanocellulose films using a roll-to-roll nanoimprinting method. *Microelectronic Engineering* **2016**, *163*, 1-6.
(4) Pöhler, T.; Mautner, A.; Aguilar-Sanchez, A.; Hansmann, B.; Kunnari, V.; Grönroos, A.; Rissanen, V.; Siqueira, G.; Mathew, A. P.; Tammelin, T. Pilot-scale modification of polyethersulfone membrane with a size and charge selective nanocellulose layer. *Separation and Purification Technology* **2022**, *285*, 120341.
(5) Kontturi, E.; Thüne, P. C.; Niemantsverdriet, J. Cellulose model surfaces simplified preparation by spin coating and characterization by X-ray photoelectron spectroscopy, infrared spectroscopy, and atomic force microscopy. *Langmuir* **2003**, *19* (14), 5735-5741.
(6) Chen, F.; Xiang, W.; Sawada, D.; Bai, L.; Hummel, M.; Sixta, H.; Budtova, T. Exploring large ductility in cellulose nanopaper combining high toughness and strength. *ACS nano* **2020**, *14* (9), 11150-11159.
